# Impacts of Surface Adsorption on Water Uptake within a Metal Organic Nanotube Material

**DOI:** 10.1021/acs.langmuir.2c01124

**Published:** 2022-11-07

**Authors:** Lindsey C. Applegate, Vidumini S. Samarasiri, Johna Leddy, Tori Z. Forbes

**Affiliations:** Department of Chemistry, University of Iowa, Iowa City, Iowa52242, United States

## Abstract

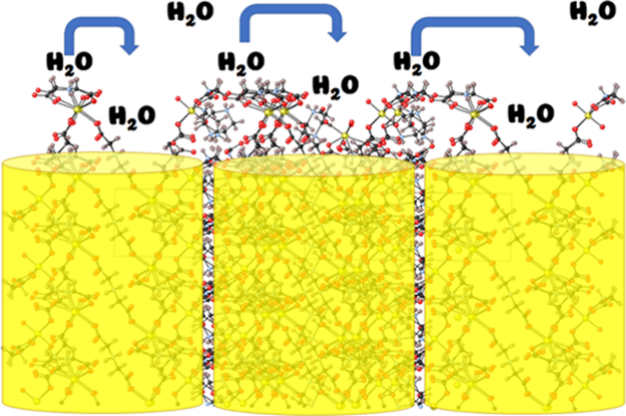

The confinement-dependent properties of solvents, particularly water, within nanoporous spaces impart unique physical and chemical behavior compared to those of the bulk. This has previously been demonstrated for a U(VI)-based metal organic nanotube that displays ice-like arrays of water molecules within the 1-D pore space and complete selectivity to H_2_O over all other solvents and isotopologues. Based upon our previous work on D_2_O and HTO adsorption processes, we suggested that the water uptake was controlled by a two-step process: (1) surface adsorption via hydrogen bonding to hydrophilic amine and carboxylic groups and (2) diffusion of the water into the hydrophobic 1-D nanochannels. The current study seeks to evaluate this hypothesis and expand our existing kinetic model for the water diffusion step to account for the initial surface adsorption process. Vapor sorption experiments, paired with thermogravimetric and Fourier-transform infrared analyses, yielded uptake data that were fit using a Langmuir model for the surface-adsorption step of the mechanism. The water adsorption curve was designated a type IV Brunauer–Emmett–Teller isotherm, which indicated that our original hypothesis was correct. Additional work with binary solvent systems enabled us to evaluate the uptake in a range of conditions and determine that the uptake is not controlled by the vapor pressure but is instead completely dependent on the relative humidity of the system.

## Introduction

Confinement of water within nanoporous spaces can lead to deviations in its chemical and physical properties that are of interest for advanced separation and storage processes. These variations arise from changes in surface area-to-volume ratios upon reduction to the nanoscale, which then cause changes in the energetics and properties of the material.^1−3^ Within synthetic and natural nanoporous materials, unusual behavior in ordering and mobility of water has been observed when the molecules are placed under nanoconfinement.^4−8^ For example, aquaporin proteins within cell membranes allow for the selective passage of water through the hydrophobic phospholipid layer by way of a single column of hydrogen-bonding water molecules.^9,10^ In addition to the high selectivity of water, these biological channels can support high water permeation rates of ∼10^–13^ cm^3^/s or three water molecules/ns for a single pore.^11^ The pore diameter of aquaporin channels ranges from 5 to 10 Å, and similar nanoconfinement effects have been observed in synthetic materials with channel diameters of 5–20 Å.^10^ Optimizing these nanoconfinement effects for these synthetic materials has important implications for applications such as catalysis, energy storage, and water purification.^12−17^

To precisely understand and therefore control the behavior of water within nanoporous spaces, it is important to consider interactions between the confined molecules and the interior pore walls both in biological and synthetic systems. The behavior of water molecules within these nanopores is largely controlled by the functionalization of the pore walls as evidenced by the nature of the aquaporin channel.^10^ As the water moves through the biological channel, asparagine residues restrain the water molecule to lie perpendicular to the channel axis. Hydrophobic amino acids (valine and leucine) prohibit different hydrogen bonding networks as the water continues along the channel and constrain the specific orientation that was initially directed by the hydrophilic group.^10^ Taking this idea to synthetic materials, a hydrophobic interior surface will promote increased solvent–solvent interactions and hydrogen bonding between the water molecules in the pores. This type of interaction can be observed in single-walled carbon nanotubes that are between 8 and 20 Å in diameter and leads to the formation of ice-like networks throughout the channel.^18−25^ Conversely, hydrophilic pores will interact more strongly with the water, which may lead to surface adsorption and changes in water mobility throughout the channel. Aluminosilicate imogolite (Al_2_SiO_3_OH_4_) contains channels that are 20 Å in diameter, and the hydrophilic nature of the channel walls leads to weak interaction with the aluminum hydroxide surface.^26^ This then leads to water clustering in the void space and dynamics that are characteristic of bulk liquid water. Mixed confinement, which consists of both hydrophilic and hydrophobic regions, would be more similar to the controls observed in aquaporin proteins, but there is limited information of confinement effects within these types of synthetic materials.

**Figure 1:**
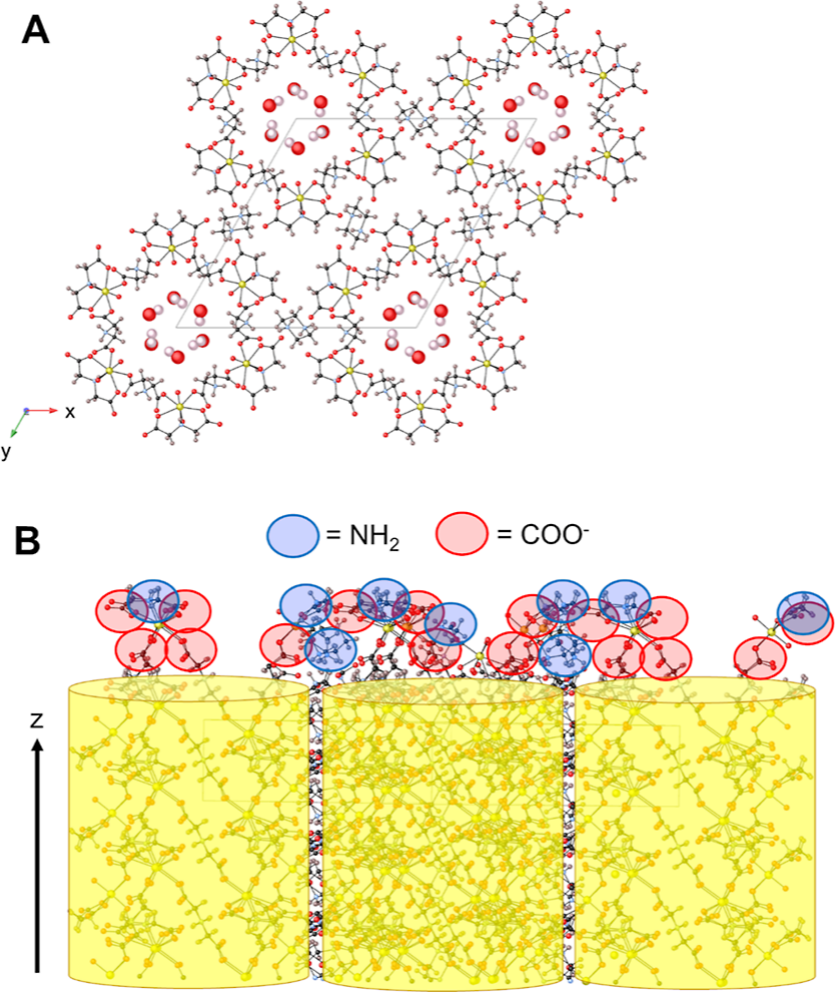
(a) Singular nanotubular unit of the UMONT material viewed down the *z*-axis that includes the internal network of water molecules. The U, O, C, N, and H atoms are depicted as yellow, red, black, blue, and pink spheres, respectively. (b) Side view of the UMONT material that demonstrates the position of the nanotubes within the crystalline lattice (yellow tubes) and hydrophilic surface groups on the exterior of the nanotube. Carboxyl oxygen atoms and protonated amines on the surface of the UMONT material are highlighted using the red and blue circles, respectively. Note: these models are not space-filling and do not accurately depict the relative size of the molecules.

Hybrid organic–inorganic materials [e.g., 3-D metal organic frameworks (MOFs), hybrid 2-D sheets, and 1-D metal organic nanotubes (MONTs)] offer a unique way to tune the structural features and functional groups within the pore space of the materials and the resulting nanoconfinement effects. The connectivity and dimensionality of these materials can be precisely controlled by tuning the organic ligand and offer expansive possibilities for precise controls,^27,28^ but literature results of nanoconfinement effects are most prevalent within one-dimensional MONTs and can be explored in depth via a selection of review articles.^29,30^ For example, Fei et al. presented a zinc-based metal–organic nanotube that confines water molecules within the pore to a single strand.^31^ This particular ordering of water molecules arises from the hydrophilic nature of the pore interior, allowing for strong hydrogen-bonding interactions with the solvent molecules.^31^ Otake et al. synthesized platinum-based MONTs with a hydrophobic pore that promotes water–water interactions. In this case, the water molecules arrange into alternating square and octameric nets within the channel of the nanotube.^32^ We have previously described and characterized a U(VI) MONT [UMONT, (pip)_0.5_[(UO_2_)(HIDA) (H_2_IDA)]·2H_2_O (pip = piperazinium; IDA = iminodiacetate)] that exhibits unique solvent uptake properties and interactions with water (Figure 1a).^33^ A UMONT is built upon U(VI) metal centers that strongly bond to two O atoms in the axial positions to create the uranyl cation (UO_2_^2+^). Six metal units are joined by IDA linkers to create corrugated macrocycles that then are stacked into nanotubular arrays connected through strong hydrogen bonding interactions. Carboxyl and protonated amine groups on the surface of the nanotube result in a hydrophilic environment on the crystal surface, but due to the poor Lewis basicity of the bound oxo groups and the position of the organic linkers, the interior pore walls of the nanotube are considered hydrophobic.^34^ Therefore, the confined water molecules interact more strongly with themselves than with the walls of the nanotube, which results in a highly ordered array that mimics the Ice-I (I_h_) structure.

To provide a deeper insight into this topic, we have previously utilized the UMONT material as a model system to explore the water diffusion, interactions, and behavior to understand structure–function relationships that control water behavior within nanopores. The diffusion coefficient for the water within the UMONT pores was previously determined to be (1.2 ± 0.6) × 10^–12^ cm^2^ s^–1^, which is an order of magnitude lower than what is observed for hydrophobic single-walled carbon nanotubes.^35^ Earlier studies have also demonstrated that the interaction between the water molecules and the interior walls of the nanotube is 7 kJ/mol, which suggests some interactions with the interior walls of the channels but much less so than would be expected for hydrophilic surfaces. Additional experiments determined that the UMONT material is completely selective to water,^36,37^ and our most recent work suggests that it is related to the hydrophilic nature of the UMONT crystal surface (Figure 1b).^38^ In this previous work, we demonstrated that in the presence of pure D_2_O (99.999%) and HTO systems, only H_2_O could be observed within the nanoporous channels. A two-step adsorption mechanism then was proposed that accounts for proton exchange to explain this behavior.^38^ This proposed mechanism consists of an initial D_2_O adsorption step to the surface of the UMONT crystal, which contains multiple exchangeable amine groups associated with the IDA linker and the piperazinium counterion. During this step, the hydrogen atoms in the surface functional groups could exchange with protons in the solvent, and once this exchange is complete, H_2_O molecules are free to diffuse into the pore space.

The current work seeks to evaluate the validity for the two-step water adsorption process for the UMONT material to further our understanding of selective water adsorption within metal organic materials. Herein, we fully evaluate the water isotherm for the UMONT material to explore the water uptake process. Based upon this information, we also measured the uptake of solvent mixtures (D_2_O/H_2_O, ethanol/H_2_O, acetic acid/H_2_O, and acetone/H_2_O) to provide insights into the driving force for the selectivity and kinetics of this process. We then evaluated the use of a Langmuirian model to describe the surface adsorption process and evaluated the thermodynamics of these UMONT surface interactions that lead to the observed uptake within this material.

## Experimental Methods

### Synthesis of the UMONT Material

The UMONT material was prepared according to a previously established synthesis.^33^ Briefly, aqueous solutions of piperazine (3 mL, 0.2 M), IDA (3 mL, 0.2 M), and uranyl nitrate hexahydrate (1.5 mL, 0.2 M) were mixed in a 20 mL scintillation vial. A 7 mL aliquot of acetone was then layered into the solution to serve as the crystallization agent. *Caution*: The synthesis of the UMONT material requires the use of radioactive ^238^U, which is an α-emitter and like all radioactive materials must be handled with care. These experiments were conducted by trained personnel in a licensed research facility with special precautions taken toward the handling, monitoring, and disposal of radioactive materials.

The vial containing the chemical reagents was left uncapped at room temperature, and large yellow crystals formed within 3–5 days at yields of >95% based upon U. Identity of the initial sample was confirmed via unit cell determination collected on a single crystal using a Bruker D8 Quest X-ray diffractometer equipped with a Mo microfocus beam (Mo Kα; λ = 0.71073 Å) and an Oxford Systems low-temperature Cryosystem operating at 100 K. Data were collected with the Bruker APEX3 software package using a unit cell scan with a 5 s exposure time over 180 frames. After identification, the crystals were lightly grouped, and bulk purity was confirmed by powder X-ray diffraction (PXRD). Approximately 20 mg of the homogenized sample was placed on a zero-background silicon wafer and measured with a Bruker D8 Advance diffractometer equipped with nickel-filtered Cu Kα radiation (λ = 1.5418 Å). Voltage and current of the instrument were set to 40 kV and 40 mA, respectively, and data were collected with a scan range of 5–40° 2θ and a step size of 0.05°. Purity was confirmed via comparison of experimental and calculated PXRD patterns (Mercury software) from the finalized crystallographic information file (CIF). Water uptake and release behavior was confirmed using a TA Instruments Q500 thermogravimetric analyzer equipped with a Thermo Scientific FTIR-evolved gas analyzer, and nitrogen carrier gas. Samples (10–20 mg) were heated over a range of 20–180 °C at a rate of 20 °C/min, and the mass loss was measured to evaluate water evacuation. The evolved gases traveled through the FTIR transfer line stabilized at 180 °C, and the spectra was collected throughout the heating time. Representative PXRD, thermogravimetric analysis (TGA), and Fourier-transform infrared (FTIR) characterization of the as-synthesized materials can be found in Figures S1–S3, respectively.

### Vapor Sorption Experiments

**Figure 2:**
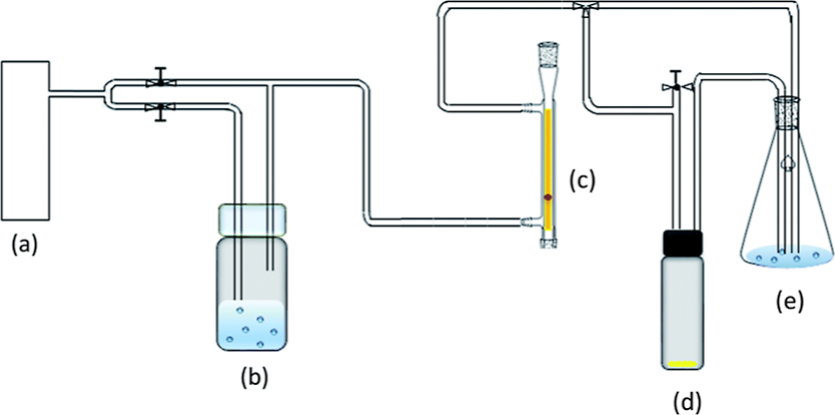
Vapor sorption apparatus. Dry nitrogen from the source (a) is bubbled through the solvent in the vessel (b). Vapor then goes through the flow meter (c) and into the sample vial (d). Bubbling is checked in the output vessel (e) to ensure that there is no blockage in the system. The valve before point (d) can be turned off to halt vapor flow to the sample vial. Figure adapted from Jayasinghe, et al.^35^ Copyright 2018 The Royal Society of Chemistry.

Characterized UMONT powder samples were dehydrated before use in the vapor sorption experiments. After confirming the purity of the crystalline UMONT compound, a 30 mg subsample of the material was dehydrated at 90 °C overnight, removed from the oven hot, placed into a 1.5 dram glass vial, and sealed with a septum cap. The starting mass of the subsample and all subsequent masses measured throughout the experiment were obtained on a Mettler AT20 microbalance, and then the vial was loaded onto the apparatus. Vapor sorption experiments were performed using a custom-built apparatus that is diagrammed in Figure 2. Dry, ultra-high-purity nitrogen gas was bubbled through a closed vessel filled with water, and the saturated vapor was mixed with dry air in varying amounts. The vapor then passes through a flow meter to regulate the amount dosed to the UMONT material, into the sample chamber, and finally to the output vessel.

Both the isotherm and the kinetic uptake experiments were performed using the vapor sorption apparatus. The isotherm data was analyzed by collecting both sorption and desorption data. Sorption experiments were conducted on the dehydrated UMONT material in a range of relative humidity values (**RH30**, **RH45**, **RH60**, and **RH80**%) to generate equilibrium mass gain of vapor into the sample. Sample weights were again measured every 10 min over the course of the 90 min experiment, with the chosen endpoint based upon observed stabilization of the sample weight. Desorption isotherms were analyzed in a similar manner, except that the as-synthesized, fully hydrated UMONT sample was used as the initial material and then subjected to the various humidity environments. Again, final masses were corrected for mass of the vial and cap and the average water adsorbed on the vial surface that was obtained from the blank. Each experiment was performed in triplicate to generate the final data points. Additional uptake kinetics were evaluated for solvent mixtures: D_2_O/H_2_O (pure—**D**_**2**_**O 100**, 90% v/v—**D**_**2**_**O 90**, 50% v/v—**D**_**2**_**O 50**), ethanol/H_2_O (90% v/v—**EtOH 90**, 80% v/v—**EtOH 80**, 50% v/v—**EtOH 50**, 10% v/v—**EtOH 10**), acetic acid/H_2_O (20% v/v—**HOAc20**), and acetone/H_2_O (50% v/v—**ACE50**). The experiments were performed in the manner described above.

### Post-Uptake Material Characterization

After each uptake experiment, the sample was immediately loaded onto a tared aluminum pan for paired TGA/FT-IR analysis. Data was collected on a TAQ500 thermogravimetric analyzer with a ramp rate of 20 °C/min from room temperature to 180 °C. Bulk purity of the UMONT material after uptake was confirmed using PXRD. Representative TGA, FTIR, and PXRD characterization of the post-uptake UMONT samples can be found in Figures S4–S10.

### Details of the Modeling Approach

A Langmuir adsorption model for these studies was developed and yielded equations for equilibrium and transient data (see Supporting Information Section 3 for additional details). An initial sample weight is determined, and then, the weight is tracked with time *t* (min). The time-dependent weight fraction *w*(*t*) is the weight at time *t* normalized by the initial sample weight, and once the weight fraction was time-invariant, the equilibrium, maximum mass of the weight fraction (*w*_max_) was determined from the model.

#### Equilibrium Data

At equilibrium, the final, maximum weight fraction *w*_max_ defines *K*[X]_g_, where *K* is the equilibrium constant for adsorption to the surface for the adsorbate (X) present in the gas phase at vapor pressure [X]_g_. As seen from Supporting Information Section 3.3.1, eq S15 specifies *K*[X]_g_ from *w*_max_.
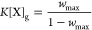
1

Vapor pressure of the adsorbate is determined from literature data^39−44^ and reported here in units of mm Hg. *K* is defined as the equilibrium constant for adsorption which is the ratio of the forward rate of adsorption, *k*_f_ [g (s mm Hg)^−1^], and dimensionless, *k*_b_, where *K* = *k*_f_/*k*_b_. The free energy of adsorption, Δ*G* (kJ mol^–1^), at temperature *T*(K) is defined using eq 2.
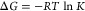
2

For pK = −log *K* and pK = Δ*G* [2.303*RT*]^−1^, *K* and Δ*G* are then determined from the steady-state data of *w*_max_ and adsorbate vapor pressure [X]_g_.

#### Transient Data

Where *w*(*t*) is known, transient data combined with *w*_max_ separates *k*_f_ and *k*_b_. Following the derivation in Supporting Information Section 3.3.2 in eqs S18–S22 leads to the following expression
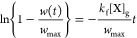
3

A plot of ln {1 – *w*(*t*)/*w*_max_} versus *t* is linear with a slope of −*k*_f_[X]_g_*w*_max_^–1^ and an intercept of 0. Defining *w*_max_ and [X]_g_ enables the value of *k*_f_ to be determined. From the steady-state data, *K* = *k*_f_/*k*_b_, so if given *K* and *k*_f_, then *k*_b_ can also be elucidated from the model.

#### Vapor Pressure for Mixtures of Organic Solvents and Water

Literature data for vapor pressure of mixtures of ethanol in water,^45^ acetone in water,^45^ acetic acid in water,^43^ and D_2_O in water^46^ are plotted against the mole fraction, X_L_, of the organic component in the liquid. For the mixtures explored in this study, moles of water (mole_water_) and the solvent (mole_solvent_) are calculated using the volume fraction, density, and molecular weight of each, and then, X_L_ can be determined from the following equation
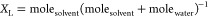
4

The vapor pressure of the solvent is extracted from the literature plots of vapor pressure with X_L_, and vapor pressure of water is defined as the product of the mole fraction of water and saturated vapor pressure of water at 25 °C and 23.76 mm Hg.

## Results and Discussion

### Water Adsorption Isotherm

**Figure 3:**
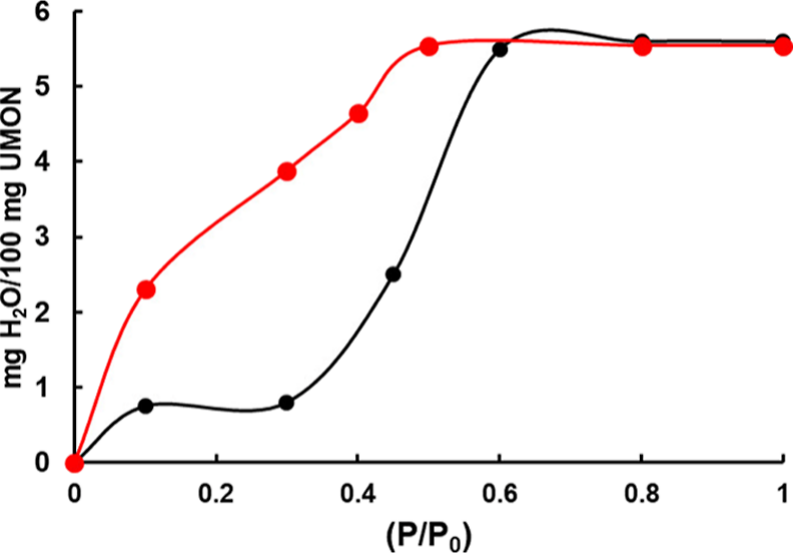
Isotherm of water vapor adsorption/desorption onto the UMONT material modeled using BET equations. The black-filled points represent adsorption, and the red points represent desorption.

The water sorption isotherm is shown in Figure 3 and depicts both the adsorption and desorption behavior of water in the UMONT material. For the adsorption process, we see minimal uptake at **RH30** and approximately 50% uptake at **RH45**. As the relative humidity increases to **RH60** and **RH80**, the UMONT material achieves complete hydration (predicted value 5.6%). For the desorption experiment, dehydration does not occur until the relative humidity decreases below 50%. The desorption process does not occur under the same conditions as those of the adsorption process as exhibited by the hysteresis loop generated upon dehydration of the UMONT material.

Brunauer–Emmett–Teller theory classifies adsorption isotherms into six main types that can provide insights into the nature of the porous materials.^47−49^ The first class (type I) is essentially a simple Langmuir adsorption model that depicts monolayer adsorption on microporous solids and assumes that the adsorption and desorption processes are completely reversible. Type II and III isotherms both can be applied to non-porous or microporous adsorbents and are also reversible adsorption processes. The difference between these two models is that type II represents a monolayer–multilayer adsorption process and exhibits a convex isotherm at low pressures, while type III represents multilayer adsorption. Types IV and V are seen with adsorbents that contain pores between 1.5 and 100 nm and occur when capillary condensation follows adsorption.^50^ The difference between types IV and V is analogous to that between types II and III. Particularly, type IV adsorption behavior is characterized by monolayer–multilayer adsorption followed by pore filling and the presence of a hysteresis loop traditionally is associated with the capillary condensation of the solvent into the pores. Type V also follows the two-step process (surface adsorption followed by pore filling), but the adsorption occurs as an unrestricted multilayer that forms because lateral interactions between adsorbed molecules are stronger than interactions between the adsorbent surface and adsorbate. For type VI, the material is non-porous and contains multiple steps that represent stepwise multilayer adsorption. The step height for this type of isotherm represents the monolayer capacity for each adsorbed layer.^51^

The shape of the isotherm associated with the UMONT system combined with our structural understanding of the pore shape is suggestive of the type IV adsorption behavior. We clearly observe an S-shaped curve that contains a hysteresis loop that would be indicative of the BET isotherms of these types. Clear differentiation between two types using our vapor apparatus is dependent on the shape of the curve at lower relative humidity values. Although we do not see the hysteresis loop close until *P*/*P*_0_ = 0, we do note that both the desorption and adsorption curves are concave, and this would signify a type IV model. Given the surface chemistry, we predict that the interactions between the protonated amines on the surface and adsorbed water molecules would be stronger than the water–water interactions, which would also favor a type IV model. This clearly fits with our initial hypothesis that the uptake process occurs by first adsorption of the water on the surface followed by pore filling after the formation of the multi-layer structure.

Water vapor isotherms for MOF materials have also been analyzed and reveal differences related to the surface chemistry and porosity of the material. The isotherms for these materials range from type I to type V and can be delineated based upon differences in structural details of the materials. Liu et al. collated isotherm data for 18 different MOF compounds and distinguished 3 categories for water adsorption in MOFs: (i) chemisorption on metal nodes; (ii) physisorption via capillary condensation; or (iii) physisorption via layer/cluster adsorption.^52^ They noted that capillary condensation can only be associated with pore diameters that are larger than a critical value (*D*_c_), which for water is approximately 2.0 nm. However, hysteresis loops within the adsorption isotherms could also be observed for MOFs with pore diameters less than that value, and so they further defined the origins of the hysteresis loop to (i) capillary condensation when pores are greater than 2.0 nm; (ii) formation of water superclusters in pores that are smaller than *D*_c_; and (iii) when the flexibility of the pore space approaches the critical value.

The largest hysteresis loops reported by Liu et al. were associated with MIL-53(Al)-OH, Cr-soc-MOF-1, and Y-shp-MOF-5.^52^ In the case of MIL-53(Al)-OH, the large hysteresis loop was associated with the flexibility of the system which causes the pore to become very close to the *D*_c_ value, as the initial pore dimensions of 1.9 nm × 0.8 nm swell to 1.7 nm × 1.2 nm.^53^ Similarly, Cr-soc-MOF-1 contains a 1-D channel with a diameter of 1.7 nm, which again is close to the *D*_c_ value.^54^ The crystal structure analysis suggested that nucleation of the water occurs on the vertices of the cubic cages, and water filling occurs between 60 and 75% relative humidity. In the case of Y-shp-MOF-5, there are 12-coordinated nonanuclear secondary building units linked via 12 BTEB units to create a 1D channel that is 1.2 nm in diameter.^55^ This material possesses an isotherm that was designated type IV with an S-shaped profile. The authors described the behavior such that at low relative humidity (10%), there is initial adsorption to open sites on the metal center and hydroxyl groups on the surface.^55^ As the relative humidity increases to 50%, there is a steep uptake due to dispersive energy of clusters of at least five water molecules, referred to as water superclusters, which is substantive enough to be maintained inside the pores. The hysteresis then is driven by the need to break these water superclusters into smaller units before escaping the pore.^52^

Our UMONT material is closely aligned with the interpretation of the adsorption isotherm associated with Y-shp-MOF-5 but has some clear differences as it relates to water ordering and selectivity. Both materials contain hexagonal/trigonal arrangements with 1-D channel sizes of 1.2 nm, which is much less than the *D*_c_ value. Both materials have similar adsorption–desorption behavior with adsorption for the UMONT material occurring at a lower relative humidity (20% compared to 30% for Y-shp-MOF-5). The initial nucleation on a hydrophilic site and the formation of superclusters composed of at least five water molecules suggested for Y-shp-MOF-5 agree well with the structural information from the UMONT material. In the UMONT, we believe that the hydrophilic surface provides the adsorption point, and then, we observe hexameric clusters of water molecules arranged into an extended network within the pore space. One major difference between the materials is that the ordered water observed for the UMONT is not maintained within the Y-shp-MOF material upon hydration, and instead, AbdulHalim et al. report relatively diffuse electron density under high-relative humidity conditions.^55^ In addition, the Y-shp-MOF-5 material is not selective to water as other solvents were initially observed within the void spaces. The selectivity of the UMONT material has been noted in a range of conditions and will be utilized in the next section to further evaluate the mechanism of uptake.^33,36,37,56^

### Multicomponent Solvent Uptake

**Figure 4:**
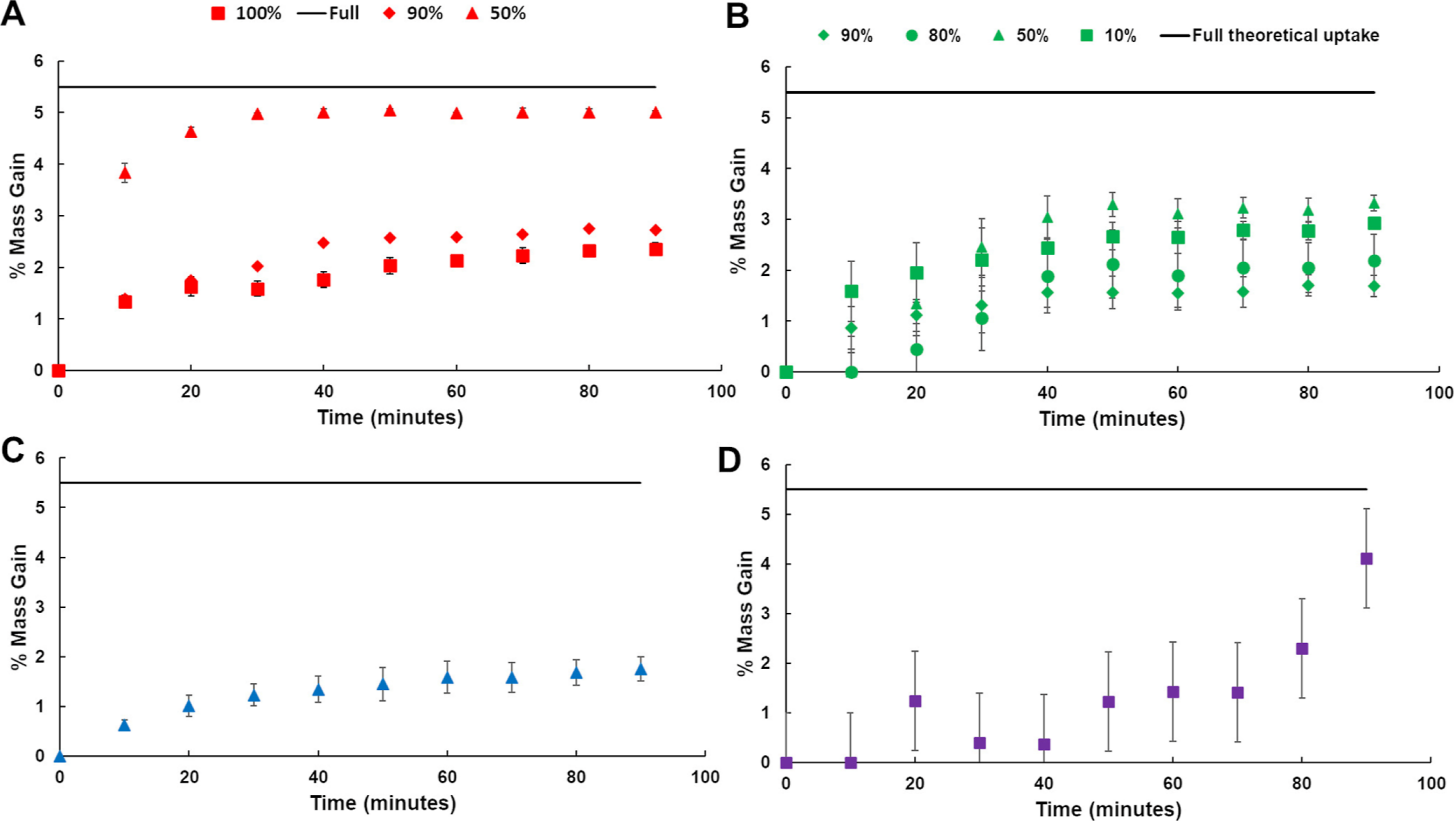
Uptake curves associated with mass gain of UMONT crystals upon exposure to solvent mixtures of (a) 100 (**D**_**2**_**O 100**), 90 (**D**_**2**_**O 90**), and 50% (**D**_**2**_**O 50**) D_2_O/H_2_O, (b) 90 (**EtOH 90**), 80 (**EtOH 80**), 50 (**EtOH 50**), and 10% (**EtOH 10**) ethanol/H_2_O, (c) 50% (**ACE 50**) acetone/H_2_O, and (d) 20% (**HOAc 20**) acetic acid/H_2_O. Full H_2_O uptake mass gain is expected at 5.6% mass gain.

We next move to binary systems to evaluate the uptake behavior and kinetics of the UMONT using the custom-built vapor sorption instrument. Uptake curves associated with D_2_O/H_2_O (pure—**D**_**2**_**O 100**, 90% v/v—**D**_**2**_**O 90**, 50% v/v—**D**_**2**_**O 50**), ethanol/H_2_O (90% v/v—**EtOH 90**, 80% v/v—**EtOH 80**, 50% v/v—**EtOH 50**, 10% v/v—**EtOH 10**), acetic acid/H_2_O (20% v/v—**HOAc 20**), and acetone/H_2_O (50% v/v—**ACE50**) are depicted in Figure 4. In all cases, we only observe H_2_O within the pore space, which was confirmed via analysis of the evolved gases produced during heating by TGA and FTIR (Figures S4–S9). In addition, we observe no degradation of the UMONT material, as evidenced by the powder X-ray diffractograms obtained after the vapor uptake experiments (Figure S10).

The uptake curves generated from D_2_O exposure were compared to those of pure H_2_O exposure (Figure 4a) and were similar to our previous study on water isotopologues. As demonstrated in previous work by Jayasinghe et al., the UMONT exposed to a saturated H_2_O environment reaches full uptake at (5.58 wt %) in less than 30 min.^35^ None of the D_2_O experiments reach full uptake after a 90 min equilibration time, which was also previously observed by Payne et al.^38^ The **D**_**2**_**O 100** and **D**_**2**_**O 90** experiments have an average maximum mass gain of 2.3 and 2.7 wt %, respectively. Payne et al. also reported lower uptake values (2.1%) for the UMONT exposed to pure D_2_O, which demonstrated the reproducibility of the data.^38^ In this previous study, we also noted that even in the presence of pure D_2_O, we observed H_2_O released as the evolved gas. Our current hypothesis for this result is that the pores are highly selective to H_2_O, so initial exchange between D_2_O and the exchangeable protons on the surface of the UMONT must occur, followed by uptake into the pore channels.^57^ The **D**_**2**_**O 50** sample exhibits a mass gain of 4.9 wt %, which suggests that 88% of the nanochannels are filled under these conditions. Water isotopologues have similar vapor pressures (H_2_O = 0.031 atm; D_2_O = 0.0269 atm); thus, we believe that the changes in uptake curve behavior are directly related to the amount of H_2_O in the system and therefore the vapor phase.^58^

**EtOH 90**, **EtOH 80**, **EtOH 50**, and **EtOH 10** were examined in a similar fashion (Figure 4b). **EtOH 90** denoted a mass gain of 1.7 wt % that increases with the increasing H_2_O content with **EtOH 80**, **EtOH 50**, and **EtOH 10** (2.2%, 3.3%, and 2.9 wt %, respectively). Even at the highest loading observed for **EtOH 10,** the mass loading of 3.3 wt % indicates that 59% of the UMONT pores are occupied by water molecules.

One acetic acid/H_2_O system (**HOAc 20**) was also evaluated, but other concentrations were not explored due to issues with material stability. Average mass gain associated with **HOAc 20** was 4.1 wt %, which indicates 71% pore filling. We note that the error associated with the vapor sorption experiments in the case of **HOAc 20** is much larger than what is observed for the other mixed solvent systems. With increasing amounts of acetic acid in the system, the UMONT crystals began to degrade and dissolve in the solution. Thus, the 20% acetic acid solution is likely the upper limit in stability for the UMONT material for this system and may experience surface degradation even at 20%, so we did not attempt higher concentrations or utilize these experiments in our modeling.

In the case of acetone (**ACE 50**), 1.75 wt % suggests only 31% filling of the available nanochannels within the UMONT material. Only one solvent ratio was attempted in this case because of concerns that acetone may be able to occupy secondary channels in the UMONT material. The presence of acetone was noted in previous work by Jayasinghe et al., but this was when acetone was used as the crystallization agent for the synthesis of the UMONT material.^35^ However, as we increased the amount of acetone in the solvent system, we noted much larger errors and unreasonable weight gains (11%); thus, we did not pursue higher acetone ratios for this system.

Total uptake for each solvent mixture is markedly lower than the full uptake (5.6%) observed for pure water and can be loosely correlated to the magnitude of vapor pressure of each solvent mixture (Table 1). In general, lower vapor pressures of the solvent correlate to increased uptake percentages in the material, and since the UMONT is completely selective to water, this behavior is analogous to the relative humidity of the sample chamber. However, there are some important distinctions that need to be considered. For example, the average mass gain for **D**_**2**_**O 90** and **EtOH 80** is similar at 2.7 and 2.2 wt %, respectively, but the vapor pressure of H_2_O under these conditions is 2.38 mm Hg for the **D**_**2**_**O 90** system and 10.6 mm Hg for **EtOH 80**. Therefore, we turn to additional modeling to explore the nature of the surface adsorption to the UMONT material.

**Table 1:** Tabulated Outcomes for Fitting of the Data Based on Analysis with Vapor Pressure [X]_g_ of the Organic Solvent

sample	*n*	*w*_max_ %	VP [X]_g_ (mmHg)	*K*	Δ*G* (kJ/mol)	*k*_f_ (/s)	*k*_b_ (/s)
D_2_O 100	3	2.36	20.57	1.18 × 10^–3^	16.7	6.43 × 10^–7^	7.38 × 10^–4^
D_2_O 90	3	2.36	20.83	1.16 × 10^–3^	16.8	6.33 × 10^–7^	8.65 × 10^–4^
D_2_O 50	3	5.05	22.13	2.41 × 10^–3^	14.9	5.34 × 10^–6^	2.22 × 10^–3^
EtOH 90	3	1.70	75.86	2.27 × 10^–4^	20.8	1.20 × 10^–7^	5.26 × 10^–4^
EtOH 80	3	2.19	72.44	4.03 × 10^–4^	19.4	2.66 × 10^–7^	6.66 × 10^–4^
EtOH 50	3	3.32	64.57	5.32 × 10^–4^	18.7	4.02 × 10^–7^	7.55 × 10^–4^
EtOH 10	3	2.96	40.74	7.48 × 10^–4^	17.8	1.07 × 10^–6^	1.43 × 10^–3^
ACE50	3	1.76	160.32	1.11 × 10^–4^	22.6	5.79 × 10^–8^	5.20 × 10^–4^

### Summary of the Langmuir Absorption Model for Surface Adsorption of the UMONT

A Langmuir absorption model was utilized to explore the UMONT uptake in a range of conditions. Data for the pure H_2_O system for four relative humidity values (**RH 30**, **RH 45**, **RH 60**, and **RH 80**) was previously collected by Jayasinghe et al. and utilized for this modeling effort.^35^ Data for mixtures of H_2_O in D_2_O are shown for D_2_O contents of 99.8, 90, and 50% as **D**_**2**_**O 100**, **D**_**2**_**O 90**, and **D**_**2**_**O 50**, respectively. Mixtures of water and organic solvents **EtOH 90, EtOH 80**, **EtOH 50**, **EtOH 10**, **HOAc 20**, and **ACE 50** were also included in the model. All mixture compositions are reported as volume percentages.

Data for *w*(*t*) with time were collected for water at various relative humidity values and for water and solvents mixed by volume as described in the Experimental Methods section. Steady-state *w*_max_ and transient *w*(*t*) were reported as weight of solvent uptake normalized by the dry particle weight. The time-dependent fraction of total uptake is reported as *w*(*t*)/*w*_max_*.* The final, equilibrium weight fraction of the adsorbate is *w*_max_, and the value varies from 0.95 to 5.65%. Data analysis is undertaken first for equilibrium *w*_max_ with vapor pressure of species X ([X]_g_) to yield the equilibrium constant for adsorption *K* and the free energy for adsorption Δ*G*. Because *K* = *k*_f_/*k*_b_, transient data determines *k*_f_ and *k*_b_, which correspond to the rates of adsorption and desorption on the surface.

**Figure 5:**
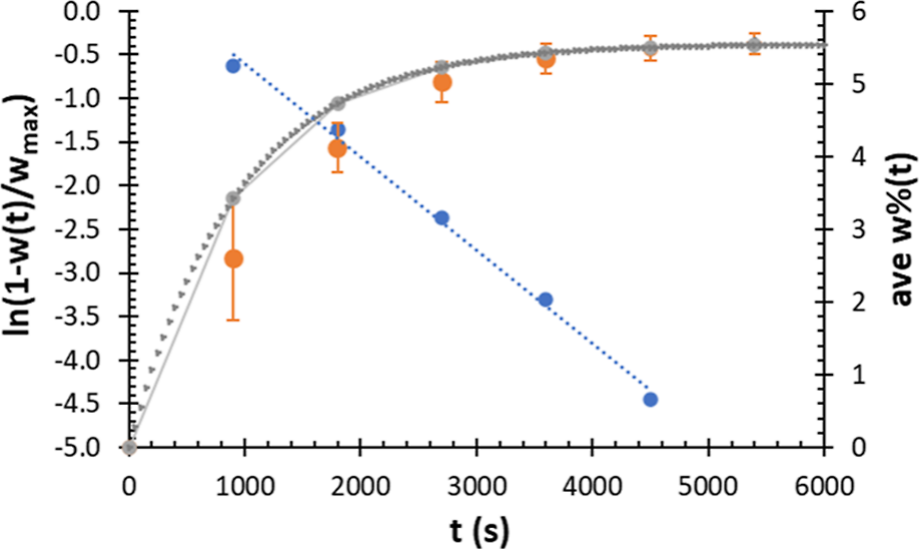
Adsorption of water to the UMONT surface for the **RH 60** environment is tracked as the average weight percent *w* %(*t*) (orange circles). Error bars are standard deviations for three replicates. As a percentage, the equilibrium *w*_max_ = 5.6%. From *w*_max_ and the vapor pressure of water [H_2_O]_g_ of 14.23 mm Hg, *K* of 4.1 × 10^–3^ and Δ*G* of 13.6 kJ mol^–1^ for water adsorption are obtained. Data are analyzed according to eq 3 over the linear range shown by blue circles. From the slope and an intercept taken as 0, the fit of *w* %(*t*) is compared, shown as gray circles and dots.

All of the data is available in the Supporting Information section (Figures S11–S19), but we will detail the data analysis for the uptake of pure water at **RH 60** as an example. Time-dependent data, *w* %(t), is analyzed according to eq 3, and ln (1 – *w*(*t*)/*w*_max_) versus time *t*(s) is plotted in Figure 5 (blue circles). The equation anticipates a linear fit with the slope of −*k*_f_[X]_g_*w*_max_^–1^, where [X]_g_ is the vapor pressure (torr) of the solvent. Regression over the linear range for this sample (900–4500 s) yields slope −(1.06 ± 0.04) × 10^–3^ and intercept 0.45 ± 0.13 with an *R*^2^ of 0.995. From the slope, *w*_max_, and eq 3, *w* %(*t*) is calculated, shown as a gray dotted line in Figure 5. The calculated *w* %(*t*) is shown as gray circles at each recording time and shows good agreement with the experimental data (orange circles). Finally, because the slope, [X]_g_, and *w*_max_ are known, *k*_f_ is calculated as 4.1 × 10^–6^ s^–1^. From *K*, the value for *k*_b_ is determined as 1.0 × 10^–3^ s^–1^. All samples were similarly analyzed, but for mixtures, analyses were done separately for vapor pressure of water [H_2_O]_g_ and vapor pressure of the solvent [X]_g_.

For the binary mixtures, the modeling results were analyzed based upon vapor pressure of the organic solvent or the vapor pressure of the H_2_O in the system to yield the *K*, Δ*G*, *k*_f_, and *k*_b_ values. Within these systems, the total vapor pressure 760 mm Hg is the sum of the vapor pressure of the solvent and the vapor pressure of water, 760 mm Hg = [H_2_O]_g_ + [X]_g_. Vapor pressure is set by the mole fraction of the component and evaluated from literature data for vapor pressure as a function of mixture composition. The model is developed for adsorption of a single species, water, or solvent, and the data fitting does not introduce species-specific information until the vapor pressure is used to extract *K* and Δ*G*. The data and values obtained from the model with regard to the vapor pressure of the organic solvent, [X]_g_, or H_2_O are provided in Tables 1 and 2, respectively.

**Table 2:** Tabulated Outcomes for Fitting of Data for 13 Systems Based on Analysis of Vapor Pressure (VP) of Water [H_2_O]_g_

sample	*n*	*w*_max_ %	VP H_2_O (mmHg)	*K*	Δ*G* (kJ/mol)	*k*_f_ (/s)	*k*_b_ (/s)
D_2_O 100	3	2.36	0.48	5.08 × 10^–2^	7.4	3.75 × 10^–5^	7.38 × 10^–4^
D_2_O 90	3	2.36	2.38	1.18 × 10^–2^	11.0	6.42 × 10^–6^	5.46 × 10^–4^
D_2_O 50	3	5.05	11.88	4.87 × 10^–3^	13.2	1.08 × 10^–6^	2.22 × 10^–4^
EtOH 90	3	1.70	6.26	2.75 × 10^–3^	14.6	1.44 × 10^–6^	5.26 × 10^–4^
EtOH 80	3	2.19	10.59	2.11 × 10^–3^	15.3	1.39 × 10^–6^	6.60 × 10^–4^
EtOH 50	3	3.32	18.13	1.89 × 10^–3^	15.5	1.43 × 10^–6^	7.55 × 10^–4^
EtOH 10	3	2.96	22.97	1.33 × 10^–3^	16.4	1.90 × 10^–6^	1.43 × 10^–3^
ACE50	3	1.76	19.09	1.16 × 10^–3^	16.8	1.57 × 10^–6^	1.36 × 10^–3^
RH 30	3	0.95	7.13	1.35 × 10^–3^	16.4	3.70 × 10^–7^	2.75 × 10^–4^
RH 45	3	3.64	10.69	3.53 × 10^–3^	14.0	1.02 × 10^–6^	2.88 × 10^–4^
RH 60	3	5.55	14.26	4.12 × 10^–3^	13.6	4.14 × 10^–6^	1.01 × 10^–3^
RH 80	3	5.65	19.01	3.15 × 10^–3^	14.3	4.40 × 10^–6^	1.40 × 10^–3^
average (no 90 or 100% D_2_O)				2.63**×**10^**–3**^	**15.2**	2.75**×**10^**–6**^	9.61**×**10^**–4**^
std dev				1.21**×**10^**–3**^	**1.3**	2.94**×**10^**–6**^	5.90**×**10^**–4**^

### Discussion of Model Outcomes Based on Vapor Pressure of Solvents

**Figure 6:**
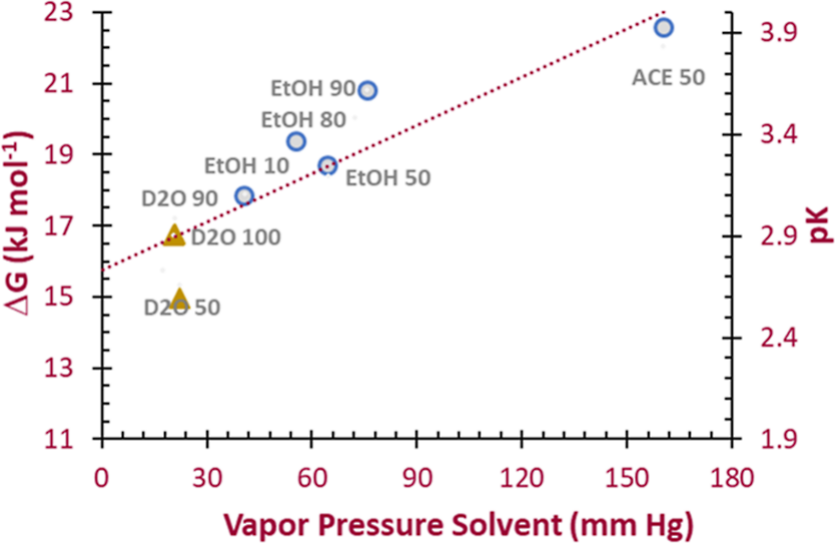
Outcomes of the model fit for the water solvent mixtures based on solvent vapor pressure. Data analyzed based on the vapor pressure of the solvent yields solvent free energy of adsorption Δ*G* and equilibrium constant pK that vary with the solvent. Crude extrapolation using a linear regression model to a system with no solvent (vapor pressure solvent = 0) estimates a Δ*G* for water of 15–16 kJ mol^–1^.

We further explore the binary systems by evaluating the free energy of adsorption Δ*G* (kJ mol^–1^) as a function of the vapor pressure of the solvent (Figure 6). Within these systems, adsorption is not favored as Δ*G* > 0 kJ mol^–1^, and the Δ*G* value tends to increase as solvent vapor pressure increases with concomitant decrease in water vapor pressure. The equilibrium constant expressed as pK = −log *K* varies in the same manner with solvent vapor pressure over the range of *K* of 1.1 × 10^–4^ to 3.5 × 10^–3^ for the solvents. Linear regression of Δ*G* with [X]_g_ is not of high quality [*R*^2^ = 0.82, Δ*G* = (0.045 ± 0.008) [X]_g_ + (15.8 ± 0.6)], but the intercept for all the solvents is 15.8 ± 0.6 kJ mol^–1^, which suggests that adsorption is dominated by the free energy of adsorption for water present in the system. Thus, the Δ*G* value at the intercept may be viewed as the component of the free energy for water adsorption, and free energy above 15.8 kJ mol^–1^ characterizes the effect of the solvent molecules on water adsorption. Overall, Δ*G* values greater than the solvent free intercept reflect the hindrance to water adsorption by solvent molecules and can be related back to the uptake of water under these different conditions.

It is important to note again that our previous data indicates that only water has been observed within the UMONT nanotubes. This was first noted by Unruh et al. where hydrated UMONT crystals were placed into dimethyl sulfoxide (DMSO) for several hours, and single-crystal diffraction and TGA of materials indicated complete dehydration but no uptake of the solvent.^36^ Only 1% mass loss was observed in this case which was correlated to surface adsorption with an uptake of 0.08 moles of DMSO/mole of the UMONT. This study was followed by work by Jayasinghe et al. who noted that uptake was not observed for any solvent but water over a range of hydrophobic and hydrophilic compounds.^35^

For the binary mixtures that include ethanol, acetone, and acetic acid, it is also important to consider the ionic interactions that occur between the solvent and the water present in the solution. We note that the vapor pressure increases as the ionic interactions between water and the solvent decrease. On one end there is acetone, the only solvent that does not ionize in water and has a substantially higher vapor pressure. Ethanol generates ions by autoprotolysis and acid–base reactions with water. The Δ*G* value calculated for the vapor pressure of the organics seems to correlate with the extent of ionization. Higher fractions of ethanol fall off this trend line, but we note that ethanol generally has higher vapor pressure and higher adsorption free energy. Both the solvent and water are likely present in the adsorbed layer, and ions, such as protons available from the surface-adsorbed solvent and water, may lower the energy of adsorption.

D_2_O/H_2_O mixtures have similar vapor pressures based on literature values, but Δ*G* varies between 15.3 and 17.2 kJ mol^–1^. Mixtures of D_2_O and H_2_O undergo isotopic exchange with a known equilibrium constant *K*_HDO_ = 3.45.
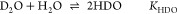


For **D**_**2**_**O 100** and **D**_**2**_**O 90** solutions, the water content is sufficiently low, and *K*_HDO_ > 1 so that the concentration of H_2_O is negligible, and the available hydrogen cations are present in HDO. For **D**_**2**_**O 50**, the concentration of H_2_O and HDO is equal. Assessment of D_2_O mixtures is complicated by the isotope exchange between D_2_O and H_2_O. Because HDO forms, adsorption of H_2_O is not the appropriate model for D_2_O mixtures with little H_2_O. As seen from the available D_2_O data, Δ*G* is lower for the highest water content, which again supports the selectivity of the H_2_O isotopologue that was previously identified by Payne et al.^38^

### Discussion of Model Outcomes Based on H_2_O Vapor Pressure

**Figure 7:**
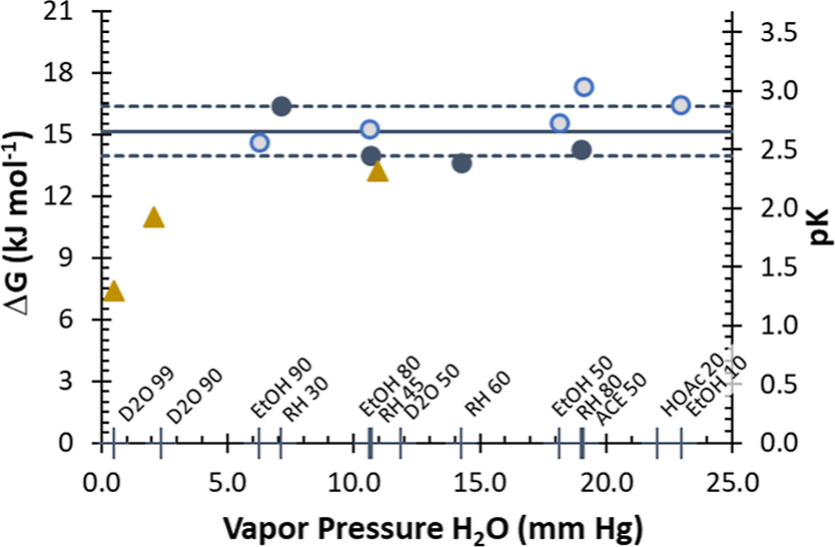
Outcomes of the model fit to the vapor pressure of water. Data fit to the vapor pressure of water yields the free energy of adsorption Δ*G* and equilibrium constant pK that are largely invariant with water vapor pressure, exclusive of **D**_**2**_**O 100** and **D**_**2**_**O 90** where HDO formation is substantial. Average Δ*G* and pK for water are 15.2 kJ mol^–1^ and 2.5 × 10^–3^, respectively, as shown on the plot.

Application of the model for vapor pressure of water yield outcomes is shown in Figure 7 and demonstrates that exclusive of the **D**_**2**_**O 100** and **D**_**2**_**O 90** data, free energy of adsorption Δ*G* for the water and the equilibrium constant pK are invariant with water vapor pressure. Average for Δ*G* is +15.2 ± 1.3 kJ mol^–1^, the same as the intercept estimated from Figure 6. In addition, pK in this case is 2.66 ± 0.21 so that *K* for water associated with the surface adsorption process is 2.5 × 10^–3^. The fact that the *K* and Δ*G* values are invariant with other solvents present suggests that the interactions of water with the UMONT surface are the process that dominates the adsorption equilibrium and kinetics. Outliers are **D**_**2**_**O 100** and **D**_**2**_**O 90**, which likely involves the isotopic exchange occurring at the surface of the material.

Several studies have explored the enthalpy (Δ*H*) of water adsorption for MOF and MONT materials, but much less is known regarding Δ*G* for this process. Grajciar et al. evaluated Δ*H* of adsorption for the CPO-27 (MOF-74) compound using density functional theory.^59^ They noted that the first and second water molecule adsorbed directly onto the dicopper paddle-wheel building unit and estimated a Δ*H*_ads_ of −49 and −44 kJ mol^–1^, respectively, where the value of −44 kJ mol^–1^ is representative of the enthalpy of condensation of water vapor to liquid.^59^ MIL-100 was explored by Jeremias et al. who found that the adsorption enthalpies decrease from −80 to −45–50 kJ mol^–1^ when the coverage increases from 0 to 20%.^58^ They suggested that this decrease in Δ*H*_ads_ supported an uptake mechanism that includes nucleation growth followed by capillary condensation. Similarly, Δ*H*_ads_ of the UMONT material has been previously characterized and also follows a multi-step process. An initial dose of 0.2 moles of water on the UMONT sample leads to a Δ*H*_ads_ of −59.8 ± 1.1 kJ mol^–1^ that then increases to −50 kJ mol^–1^ between a water dosage of 0.3 and 1.75 moles, which is representative of additional pore filling.^59^ Finally, there is a third change in the Δ*H*_ads_ value to −44 kJ mol^–1^ above two moles of H_2_O, which is when the UMONT material is completely hydrated. These studies demonstrate that overall, the water adsorption process within these porous hybrid materials is exothermic and favorable.

The positive Δ*G* observed in this study then suggests that the entropy term in the equation is negative under these conditions. Saha and Chowdhury indicate that a negative value of the Δ*S* term suggests that the adsorption process occurs by an associative mechanism.^60^ An associative mechanism occurs when there is an ordering of the water molecules on the surface through formation of a complex between the adsorbate and adsorbent. In addition, the negative value indicates that there is no significant change occurring in the internal structure of the adsorbent during the adsorption process. This agrees with our understanding of the adsorption process for the UMONT phase because we note that hydrogen bonding interactions occurring at the surface would form a more ordered water structure compared to the bulk phase, and we also observe no structural change for the material based upon our detailed analysis by XRD.

**Figure 8:**
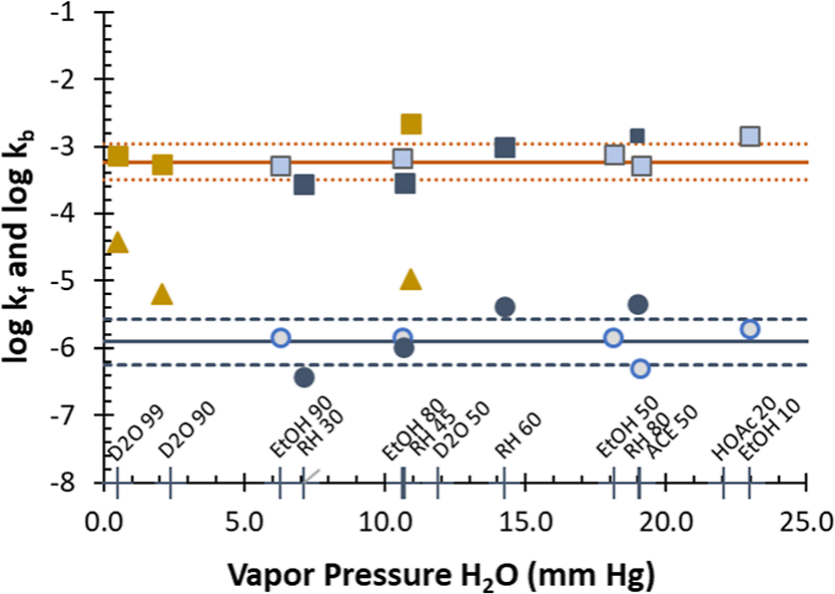
Rates of adsorption *k*_f_ and desorption *k*_b_ are invariant with vapor pressure of water, except for adsorption for **D**_**2**_**O 100** and **D**_**2**_**O 90**. Desorption is faster for water than adsorption.

Kinetic rates for adsorption k_f_ and desorption *k*_b_ were also determined from time-dependent *w*(*t*)*.* The slope determined from the plot of eq 3 determines *k*_f_ which, combined with *K* = *k*_f_/*k*_b_, determines *k*_b_. Plots of log *k*_f_ and log *k*_b_ with [H_2_O]_g_ are shown in Figure 8. For adsorption exclusive of **D**_**2**_**O 100** and **D**_**2**_**O 90**, the average rate of water adsorption *k*_f_ is (2.6 ± 2.8) × 10^–6^ (s torr)^−1^. The rate of desorption for all the solvents including **D**_**2**_**O 100** and **D**_**2**_**O 90** is (9.2 ± 5.8) × 10^–4^ s^–1^. Because *K* is the ratio of *k*_f_ to *k*_b_, *K* < 1 indicates that water adsorption is slower than and less favored than desorption. This also fits with our previous isotherm data (Figure 3) showing the hysteresis that has higher water content within the desorption steps at the same relative humidity values. As seen from Figure 3, the ratio for the pressure for the adsorption to the pressure for desorption estimates *K* as 1.6 (0.46/0.28) at 50% of the total water uptake, which is within the average *K* range of 2.5 ± 1.3 (Table 2) determined from the time-dependent adsorption data.

## Conclusions

A proposed two-step surface adsorption mechanism for selective H_2_O uptake into the UMONT material has been examined using a Langmuir adsorption model for data associated with experimental masses of crystals upon exposure to a variety of solvents and solvent mixtures. This two-step mechanism has been supported by the water adsorption isotherm that includes a hysteresis loop supportive of a type IV isotherm. Uptake of water in mixed solvent systems was evaluated using a custom-built vapor sorption apparatus and utilized to understand Δ*G*, *K*, and *k*_f_/*k*_b_ of the reaction. Uptake is dependent on the H_2_O concentration in the vapor environment, and desorption is slower than the adsorption process. Overall, the outcomes suggest that the solvent contributes to the free energy of adsorption in the binary system, but water adsorption dominates the response. In most cases, the solvent hinders water adsorption to the extent of a few kJ mol^–1^, and the positive value associated with Δ*G* of water adsorption on the surface of the UMONT suggests an associative mechanism for adsorption related to the presence of strong hydrogen bonding interactions. Future studies include functionalizing the surface of the nanotube with hydrophobic groups to alter the surface adsorption and further explore the impacts on water uptake and selectivity properties for hybrid materials.

## Supporting Information Available

PXRD, TGA, and FTIR of evolved gasses for pre- and post-adsorption and modeling details and fits for all data (PDF)

## References

[ref1] Cao G., Nanostructures and Nanomaterials, 2nd ed.; World Scientific Series in Nanoscience and Nanotechnology; World Scientific Publishing: Singapore, 2011; Vol. 2.

[ref2] Ozin G. A. Nanochemistry: Synthesis in diminishing dimensions. Adv. Mater. 1992, 4, 612–649. 10.1002/adma.19920041003.

[ref3] Malouche A.; Blanita G.; Lupu D.; Bourgon J.; Nelayah J.; Zlotea C. Hydrogen absorption in 1 nm Pd clusters confined in MIL-101(Cr). J. Mater. Chem. A 2017, 5, 23043–23052. 10.1039/c7ta07159k.

[ref4] Chemerisov S. D.; Trifunac A. D. Probing nanoconfined water in zeolite cages: H atom dynamics and spectroscopy. Chem. Phys. Lett. 2001, 347, 65–72. 10.1016/s0009-2614(01)01009-0.

[ref5] Gao Z. H.; Giovambattista N.; Sahin O. Phase Diagram of Water Confined by Graphene. Sci. Rep. 2018, 8, 6228 10.1038/s41598-018-24358-3. 29670160 PMC5906694

[ref6] Hu Y.; Bragança A. M.; Verstraete L.; Ivasenko O.; Hirsch B. E.; Tahara K.; Tobe Y.; De Feyter S. Phase Selectivity Triggered by Nanoconfinement: The Impact of Corral Dimensions. Chem. Commun. 2019, 55, 2226–2229. 10.1039/c8cc08602h. 30706910

[ref7] Krott L. B.; Bordin J. R.; Barbosa M. C. New Structural Anomaly Induced by Nanoconfinement. J. Phys. Chem. B 2015, 119, 291–300. 10.1021/jp510561t. 25494049

[ref8] Chakraborty S.; Kumar H.; Dasgupta C.; Maiti P. K. Confined Water: Structure, Dynamics, and Thermodynamics. Acc. Chem. Res. 2017, 50, 2139–2146. 10.1021/acs.accounts.6b00617. 28809537

[ref9] Jung J. S.; Preston G. M.; Smith B. L.; Guggino W. B.; Agre P. Molecular domains of the water pathway through aquaporin CHIP - The hourglass water channel model. Biophys. J. 1994, 269, 14648. 7514176

[ref10] Tajkhorshid E.; Nollert P.; Jensen M. Ø.; Miercke J. W. M.; O’Connell J.; Stroud R. M.; Schulten K. Control of the Selectivity of the Aquaporin Water Channel Family by Global Orientational Tuning. Science 2002, 296, 525–530. 10.1126/science.1067778. 11964478

[ref11] Horner A.; Siligan C.; Cornean A.; Pohl P. Positively charged residues at the channel mouth boost single-file water flow. Faraday Discuss 2018, 209, 55–65. 10.1039/c8fd00050f. 29972179 PMC6161257

[ref12] Chang C.-W.; Gong Z.-J.; Huang N.-C.; Wang C.-Y.; Yu W.-Y. MgO nanoparticles confined in ZIF-8 as acid-base bifunctional catalysts for enhanced glycerol carbonate production from transesterification of glycerol and dimethyl carbonate. Catal. Today 2020, 351, 21–29. 10.1016/j.cattod.2019.03.007.

[ref13] Chen T.; Huo P.; Hou J.-L.; Xu J.; Zhu Q.-Y.; Dai J. Confinement Effects of Metal–Organic Framework on the Formation of Charge-Transfer Tetrathiafulvalene Dimers. Inorg. Chem. 2016, 55, 12758–12765. 10.1021/acs.inorgchem.6b02062. 27989159

[ref14] de Jongh P. E.; Allendorf M.; Vajo J. J.; Zlotea C. Nanoconfined light metal hydrides for reversible hydrogen storage. MRS Bull. 2013, 38, 488–494. 10.1557/mrs.2013.108.

[ref15] Stavila V.; Bhakta R. K.; Alam T. M.; Majzoub E. H.; Allendorf M. D. Reversible Hydrogen Storage by NaAlH4 Confined within a Titanium-Functionalized MOF-74(Mg) Nanoreactor. ACS Nano 2012, 6, 9807–9817. 10.1021/nn304514c. 23075161

[ref16] Ahsan A.; Mousavi S. F.; Nijs T.; Nowakowska S.; Popova O.; Wäckerlin A.; Björk J.; Gade L. H.; Jung T. A. Phase Transitions in Confinements: Controlling Solid to Fluid Transitions of Xenon Atoms in an On-Surface Network. Small 2019, 15, 1803169 10.1002/smll.201803169. 30556276

[ref17] Boulé R.; Roiland C.; Bataille T.; Le Pollés L.; Audebrand N.; Ghoufi A. Anomalous Dynamics of a Nanoconfined Gas in a Soft Metal–Organics Framework. J. Phys. Chem. Lett. 2019, 10, 1698–1708. 10.1021/acs.jpclett.9b00421. 30913385

[ref18] Hanami K.-i.; Umesaki T.; Matsuda K.; Miyata Y.; Kataura H.; Okabe Y.; Maniwa Y. One-Dimensional Oxygen and Helical Oxygen Nanotubes inside Carbon Nanotubes. J. Phys. Soc. Jpn. 2010, 79, 023601 10.1143/jpsj.79.023601.

[ref19] Holt J. K.; Park H. G.; Wang Y.; Stadermann M.; Artyukhin A. B.; Grigoropoulos C. P.; Noy A.; Bakajin O. Fast mass transport through sub-2-nanometer carbon nanotubes. Science 2006, 312, 1034–1037. 10.1126/science.1126298. 16709781

[ref20] Giri A. K.; Teixeira F.; Cordeiro M. Structure and kinetics of water in highly confined conditions: A molecular dynamics simulation study. J. Mol. Liq. 2018, 268, 625–636. 10.1016/j.molliq.2018.07.083.

[ref21] Köhler M. H.; Bordin J. R.; da Silva L. B.; Barbosa M. C. Structure and dynamics of water inside hydrophobic and hydrophilic nanotubes. Physica A 2018, 490, 331–337. 10.1016/j.physa.2017.08.030.

[ref22] Köhler M. H.; Bordin J. R.; de Matos C. F.; Barbosa M. C. Water in nanotubes: The surface effect. Chem. Eng. Sci. 2019, 203, 54–67. 10.1016/j.ces.2019.03.062.

[ref23] Kyakuno H.; Fukasawa M.; Ichimura R.; Matsuda K.; Nakai Y.; Miyata Y.; Saito T.; Maniwa Y. Diameter-dependent hydrophobicity in carbon nanotubes. J. Chem. Phys. 2016, 145, 064514 10.1063/1.4960609.

[ref24] Kyakuno H.; Matsuda K.; Yahiro H.; Inami Y.; Fukuoka T.; Miyata Y.; Yanagi K.; Maniwa Y.; Kataura H.; Saito T.; Yumura M.; Iijima S. Confined water inside single-walled carbon nanotubes: Global phase diagram and effect of finite length. J. Chem. Phys. 2011, 134, 244501 10.1063/1.3593064. 21721637

[ref25] Maniwa Y.; Kataura H.; Abe M.; Udaka A.; Suzuki S.; Achiba Y.; Kira H.; Matsuda K.; Kadowaki H.; Okabe Y. Ordered water inside carbon nanotubes: formation of pentagonal to octagonal ice-nanotubes. Chem. Phys. Lett. 2005, 401, 534–538. 10.1016/j.cplett.2004.11.112.

[ref26] Creton B.; Bougeard D.; Smirnov K. S.; Guilment J.; Poncelet O. Molecular dynamics study of hydrated imogolite 2. Structure and dynamics of confined water. Phys. Chem. Chem. Phys. 2008, 10, 4879–4888. 10.1039/b803479f. 18688532

[ref27] Du M.; Li C. P.; Liu C. S.; Fang S. M. Design and construction of coordination polymers with mixed-ligand synthetic strategy. Coord. Chem. Rev. 2013, 257, 1282–1305. 10.1016/j.ccr.2012.10.002.

[ref28] Bosch M.; Yuan S.; Rutledge W.; Zhou H. C. Stepwise Synthesis of Metal-Organic Frameworks. Acc. Chem. Res. 2017, 50, 857–865. 10.1021/acs.accounts.6b00457. 28350434

[ref29] Applegate L. C.; Forbes T. Z. Controlling water structure and behavior: design principles from metal organic nanotubular materials. CrystEngComm 2020, 22, 3406–3418. 10.1039/d0ce00331j.

[ref30] Jia J.-G.; Zheng L.-M. Metal-organic nanotubes: Designs, structures and functions. Coord. Chem. Rev. 2020, 403, 213083 10.1016/j.ccr.2019.213083.

[ref31] Fei Z. F.; Zhao D. B.; Geldbach T. J.; Scopelliti R.; Dyson P. J.; Antonijevic S.; Bodenhausen G. A synthetic zwitterionic water channel: Characterization in the solid state by X-ray crystallography and NMR spectroscopy. Angew. Chem., Int. Ed. 2005, 44, 5720–5725. 10.1002/anie.200500207. 16059949

[ref32] Otake K.-i.; Otsubo K.; Komatsu T.; Dekura S.; Taylor J. M.; Ikeda R.; Sugimoto K.; Fujiwara A.; Chou C.-P.; Sakti A. W.; Nishimura Y.; Nakai H.; Kitagawa H. Confined water-mediated high proton conduction in hydrophobic channel of a synthetic nanotube. Nat. Commun. 2020, 11, 843–845. 10.1038/s41467-020-14627-z. 32071299 PMC7029035

[ref33] Unruh D. K.; Gojdas K.; Libo A.; Forbes T. Z. Development of Metal–Organic Nanotubes Exhibiting Low-Temperature, Reversible Exchange of Confined “Ice Channels”. J. Am. Chem. Soc. 2013, 135, 7398–7401. 10.1021/ja400303f. 23642137

[ref34] Payne M. K.; Pyrch M. M.; Jubinsky M.; Basile M. C.; Forbes T. Z. Impacts of oxo interactions within actinyl metal organic materials: highlight on thermal expansion behaviour. Chem. Commun. 2018, 54, 10828–10831. 10.1039/c8cc05240a. 30137085

[ref35] Jayasinghe A. S.; Payne M. K.; Unruh D. K.; Johns A.; Leddy J.; Forbes T. Z. Diffusion and selectivity of water confined within metal–organic nanotubes. J. Mater. Chem. A 2018, 6, 1531–1539. 10.1039/c7ta06741k.

[ref36] Jayasinghe A. S.; Salzman S.; Forbes T. Z. Metal Substitution into Metal Organic Nanotubes: Impact on Solvent Uptake and Stability. Cryst. Growth Des. 2016, 16, 7058–7066. 10.1021/acs.cgd.6b01268.

[ref37] Jayasinghe A. S.; Unruh D. K.; Kral A.; Libo A.; Forbes T. Z. Structural Features in Metal-Organic Nanotube Crystals That Influence Stability and Solvent Uptake. Cryst. Growth Des. 2015, 15, 4062–4070. 10.1021/acs.cgd.5b00653.

[ref38] Payne M. K.; Applegate L. C.; Singh P.; Jayasinghe A. S.; Crull G. B.; Grafton A. B.; Cheatum C. M.; Forbes T. Z. Selectivity for water isotopologues within metal organic nanotubes. RSC Adv. 2021, 11, 16706–16710. 10.1039/d1ra00602a. 35479164 PMC9032102

[ref39] Water Vapor Calculators. http://www.respirometry.org/calculator/water-vapor-calculators. (accessed Dec 6, 2021).

[ref40] Matsunaga N.; Nagashima A. Saturation vapor pressure and critical constants of H_2_O, D_2_O, T_2_O, and their isotopic mixtures. Int. J. Thermophys. 1987, 8, 681–694. 10.1007/bf00500788.

[ref41] Water properties. http://www.idc-online.com/technical_references/pdfs/chemical_engineering/Water_properties.pdf (accessed Dec 6, 2021).

[ref42] Makarov M. S.; Makarova S. N. Heat and mass transfer in the boundary layer during evaporation of aqueous solutions of ethanol and acetone into air and superheated vapor components of solutions. J. Phys.: Conf. Ser. 2019, 1369, 012056 10.1088/1742-6596/1369/1/012056.

[ref43] Breil M. P.; Kontogeorgis G. M.; Behrens P. K.; Michelsen M. L. Modeling of the Thermodynamics of the Acetic Acid–Water Mixture Using the Cubic-Plus-Association Equation of State. Ind. Eng. Chem. Res. 2011, 50, 5795–5805. 10.1021/ie102105r.

[ref44] Liquids. https://www.engineeringtoolbox.com/vapor-pressure-d_312.html (accessed Dec 6, 2021).

[ref45] Liu C.; Bonaccurso E.; Butt H.-J. Evaporation of sessile water/ethanol drops in controlled environments. Phys. Chem. Chem. Phys. 2008, 10, 7150–7157. 10.1039/B808258H. 19039349

[ref46] “CRC Handbook of Chemistry and Physics, 102nd Ed.”; Rumble J. R., Ed.; Taylor and Franscis: Oxfordshire, U.K., 2021.

[ref47] Kruk M.; Jaroniec M. Gas Adsorption Characterization of Ordered Organic–Inorganic Nanocomposite Materials. Chem. Mater. 2001, 13, 3169–3183. 10.1021/cm0101069.

[ref48] Barron P. M. V. R. Physical Methods in Chemistry and Nano Science; CNX, O., 2019.

[ref49] Ambroz F.; Macdonald T. J.; Martis V.; Parkin I. P. Evaluation of the BET Theory for the Characterization of Meso and Microporous MOFs. Small Methods 2018, 2, 1800173 10.1002/smtd.201800173.

[ref50] Thommes M.; Kaneko K.; Neimark A. V.; Olivier J. P.; Rodriguez-Reinoso F.; Rouquerol J.; Sing K. S. W. Physisorption of gases, with special reference to the evaluation of surface area and pore size distribution (IUPAC Technical Report). Pure Appl. Chem. 2015, 87, 1051–1069. 10.1515/pac-2014-1117.

[ref51] Sing K. S. W. Reporting physisorption data for gas/solid systems with special reference to the determination of surface area and porosity (Recommendations 1984). Pure Appl. Chem. 1985, 57, 603–619. 10.1351/pac198557040603.

[ref52] Liu X.; Wang X.; Kapteijn F. Water and Metal–Organic Frameworks: From Interaction toward Utilization. Chem. Rev. 2020, 120, 8303–8377. 10.1021/acs.chemrev.9b00746. 32412734 PMC7453405

[ref53] Shigematsu A.; Yamada T.; Kitagawa H. Wide control of proton conductivity in porous coordination polymers. J. Am. Chem. Soc. 2011, 133, 2034–2036. 10.1021/ja109810w. 21284399

[ref54] Towsif Abtab S. M.; Alezi D.; Bhatt P. M.; Shkurenko A.; Belmabkhout Y.; Aggarwal H.; Weseliński Ł. J.; Alsadun N.; Samin U.; Hedhili M.; Eddaoudi M. Reticular Chemistry in Action: A Hydrolytically Stable MOF Capturing Twice Its Weight in Adsorbed Water. Chem 2018, 4, 94–105. 10.1016/j.chempr.2017.11.005.

[ref55] AbdulHalim R. G.; Bhatt P. M.; Belmabkhout Y.; Shkurenko A.; Adil K.; Barbour L. J.; Eddaoudi M. A Fine-Tuned Metal–Organic Framework for Autonomous Indoor Moisture Control. J. Am. Chem. Soc. 2017, 139, 10715–10722. 10.1021/jacs.7b04132. 28661666

[ref56] Besley L.; Bottomley G. A. Vapour pressure of normal and heavy water from 273.15 to 298.15 K. J. Chem. Therm. 1973, 5, 397–410. 10.1016/s0021-9614(73)80031-x.

[ref57] Grajciar L.; Bludský O.; Nachtigall P. Water Adsorption on Coordinatively Unsaturated Sites in CuBTC MOF. J. Phys. Chem. Lett. 2010, 1, 3354–3359. 10.1021/jz101378z. 22170696

[ref58] Jeremias F.; Khutia A.; Henninger S. K.; Janiak C. MIL-100(Al, Fe) as water adsorbents for heat transformation purposes—a promising application. J. Mater. Chem. 2012, 22, 10148–10151. 10.1039/c2jm15615f.

[ref59] Sahu S. K.; Unruh D. K.; Forbes T. Z.; Navrotsky A. Energetics of Formation and Hydration of a Porous Metal Organic Nanotube. Chem. Mater. 2014, 26, 5105–5112. 10.1021/cm5024053.

[ref60] Saha P.; Chowdhury S. Insight Into Adsorption Thermodynamics; IntechOpen; Thermodynamics, Tadashi M., Eds., 2011.

