# Supplementary material for: Impacts of Surface Adsorption on Water Uptake within a Metal Organic Nanotube Material
Source: Langmuir. 2022 Nov 7;38(46):14025–35. doi: 10.1021/acs.langmuir.2c01124 (PMC9686127; doi:10.1021/acs.langmuir.2c01124)
Supplement: Supplementary file 1 — la2c01124_si_001.pdf [file la2c01124_si_001.pdf]

Supporting information Section

Impacts of surface adsorption on water uptake within a metal organic nanotube (MONT) material

Lindsey C. Applegate, Vidumini S. Samarasiri, Johna Leddy, and Tori Z. Forbes*

Department of Chemistry, University of Iowa, Iowa City, IA 52242

*corresponding author; tori-forbes@uiowa.edu

Table of Contents

I. **Representative characterization of the UMONT material before uptake**
II. **Representative characterization of UMONT material after uptake of binary systems**
III. **Model Description**
   1. **Characteristics for Langmuirian Adsorption**
   2. **Rate Expressions for Langmurian Adsorptions**
   3. **Simple Langmurian Model for Surface Adsorption – One Adsorbate**
IV. **Data fitting for H_2O and binary systems**
V. **References**

I. **Representative characterization of the UMONT material before uptake**

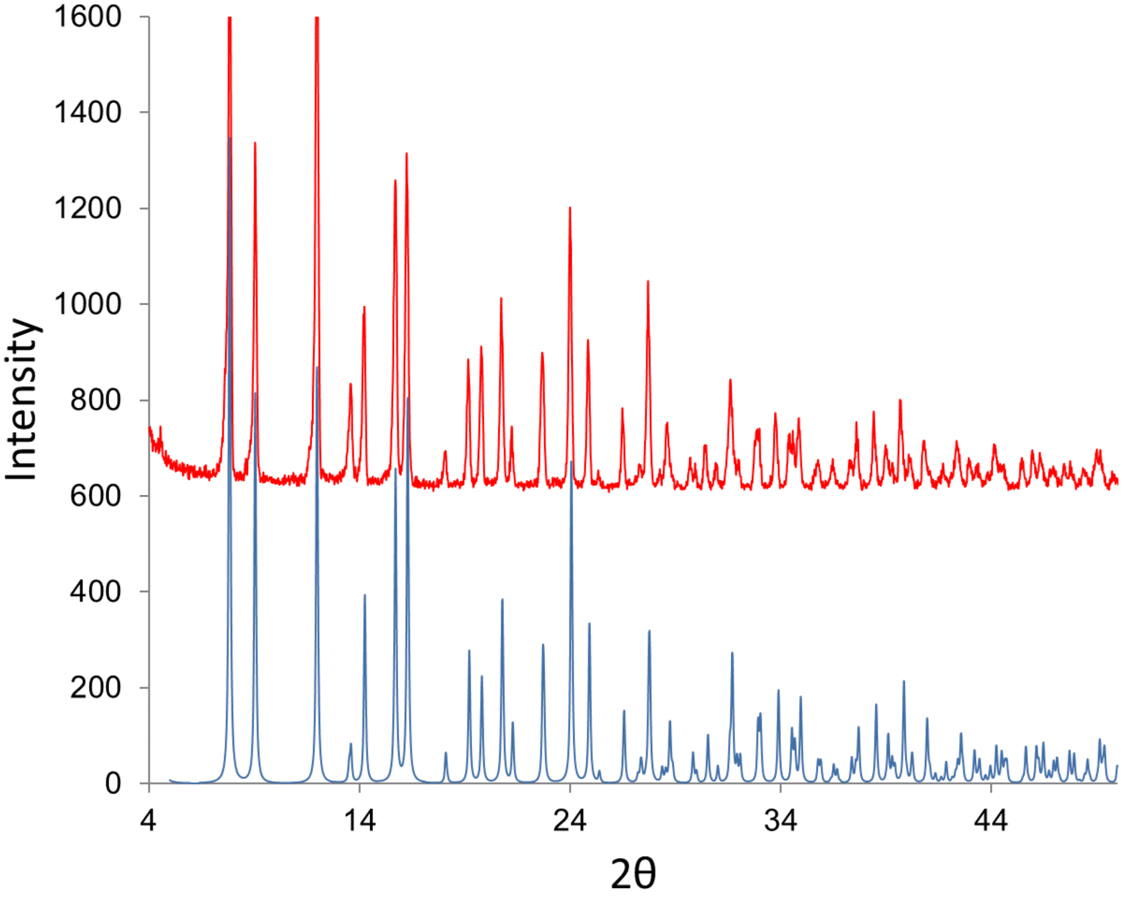

Figure S1. Representative PXRD of UMONT material post-synthesis (red) as compared to the calculated PXRD of pure material (blue).

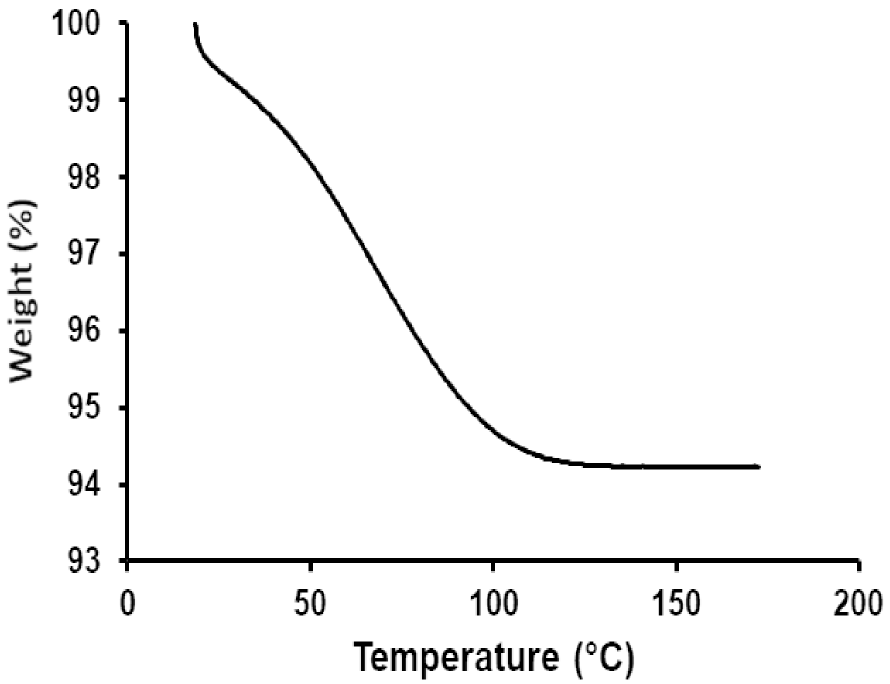

Figure S2. Representative thermogravimetric analysis (TGA) curve of UMONT post-synthesis.

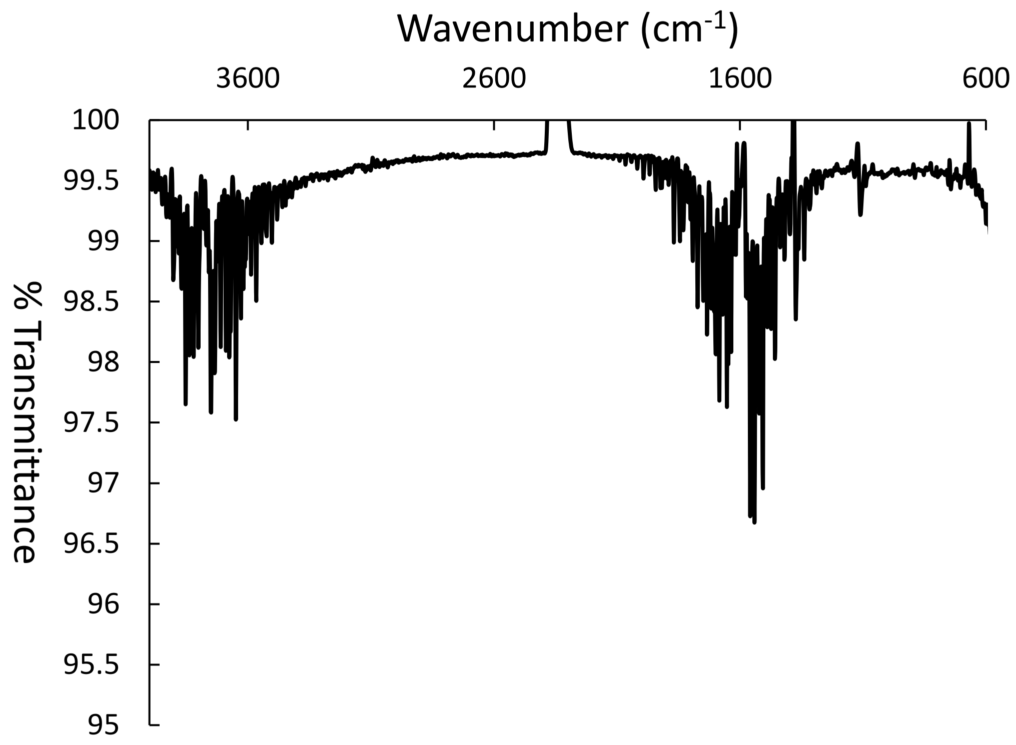

Figure S3. Representative FTIR spectrum of the evolved gas produced from the TGA of the as-synthesized UMONT material.

II. **Representative characterization of UMONT material after uptake of binary systems**

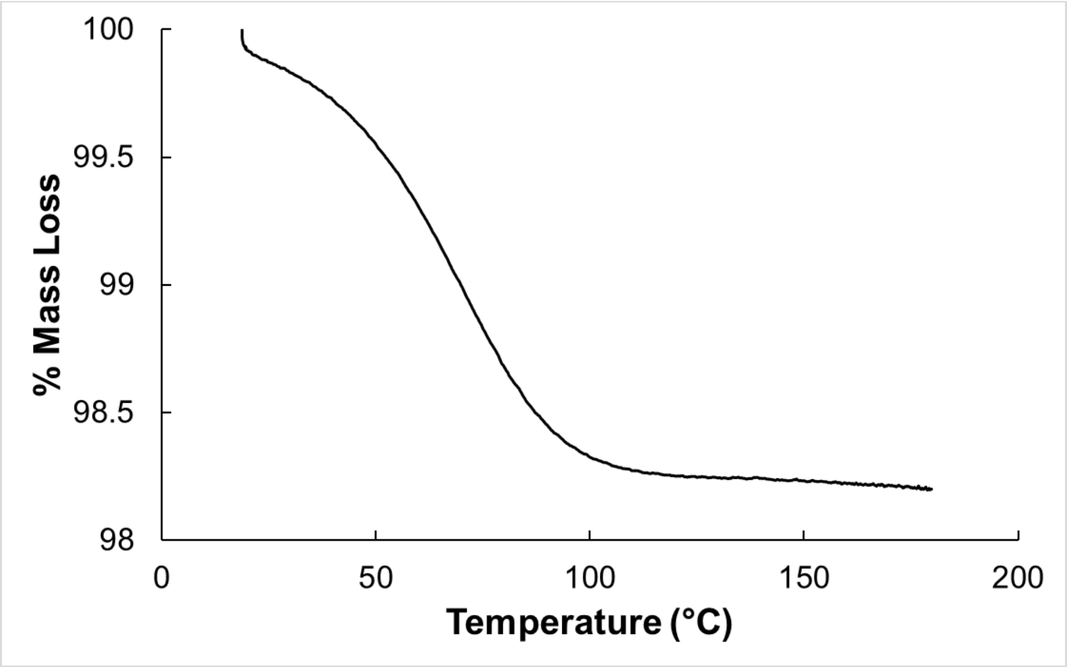

Figure S4. Representative TGA curve for **ETOH 80** system where mass gain was measured at 1.8%.

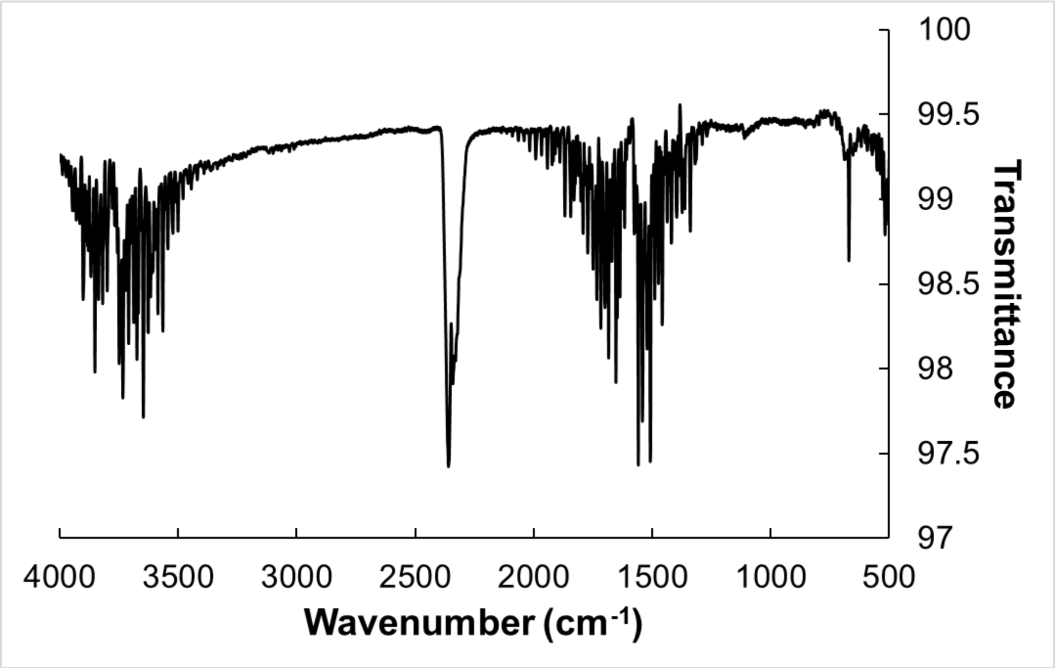

Figure S5. Representative FTIR for the evolved gas from the TGA of the **ETOH 80** system indicating that water is the only solvent present in the UMONT material.

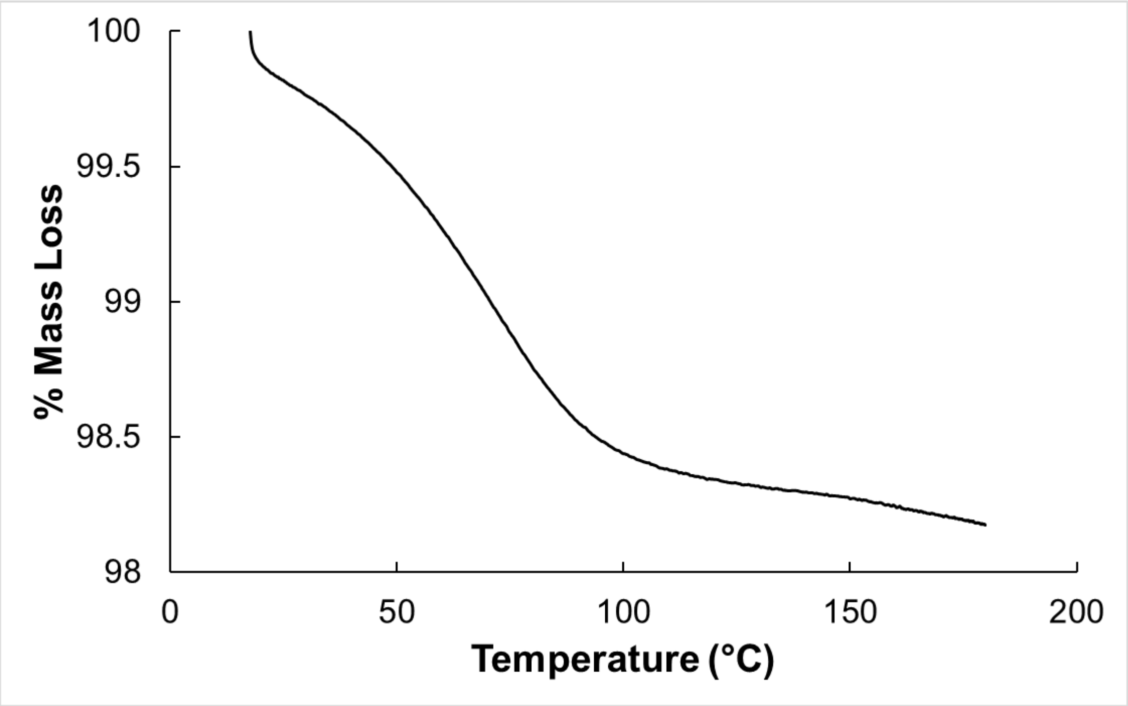

Figure S6. Representative TGA curve for **ACE50** system where mass gain was measured at 1.8%.

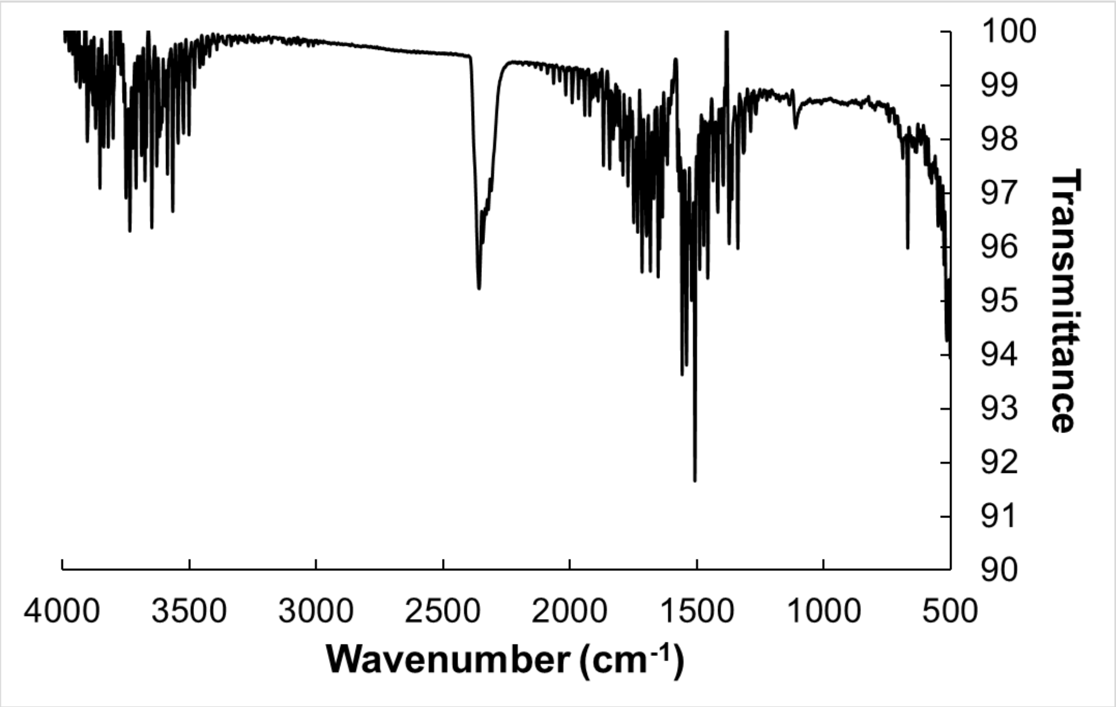

Figure S7. Representative FTIR for the evolved gas from the TGA of the **ACE50** system indicating that water is the only solvent present in the UMONT material.

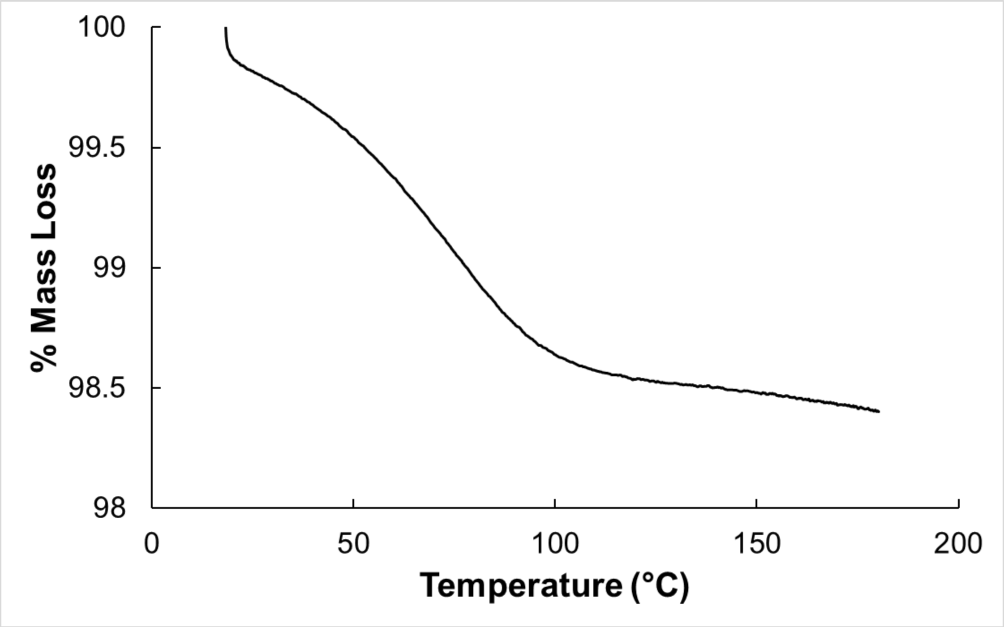

Figure S8. Representative TGA curve for **HOAc20** system where mass gain was measured at 1.59%.

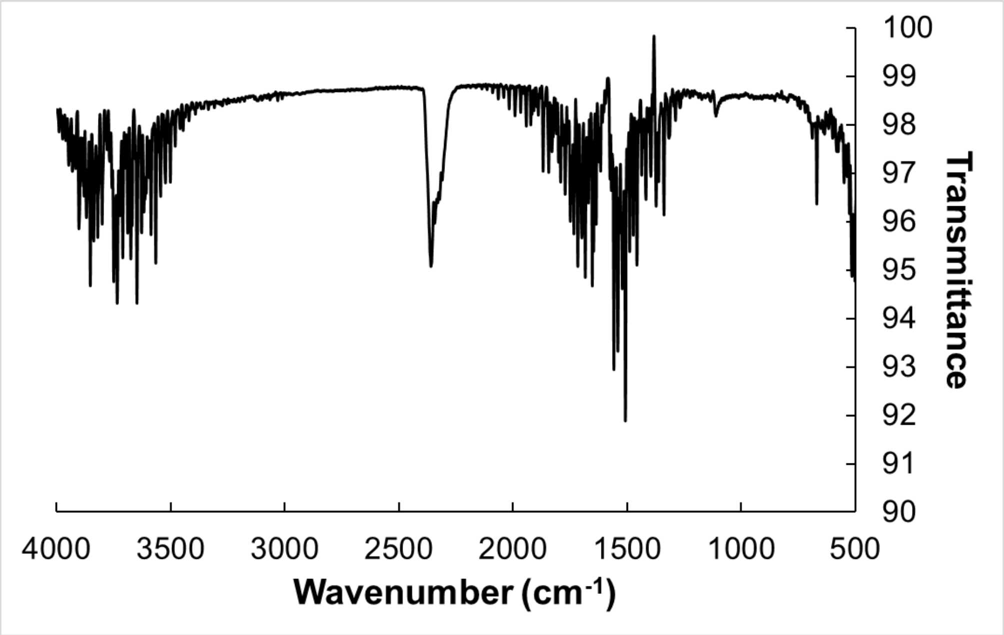

Figure S9. Representative FTIR for the evolved gas from the TGA of the **HOAc20** system indicating that water is the only solvent present in the UMONT material.

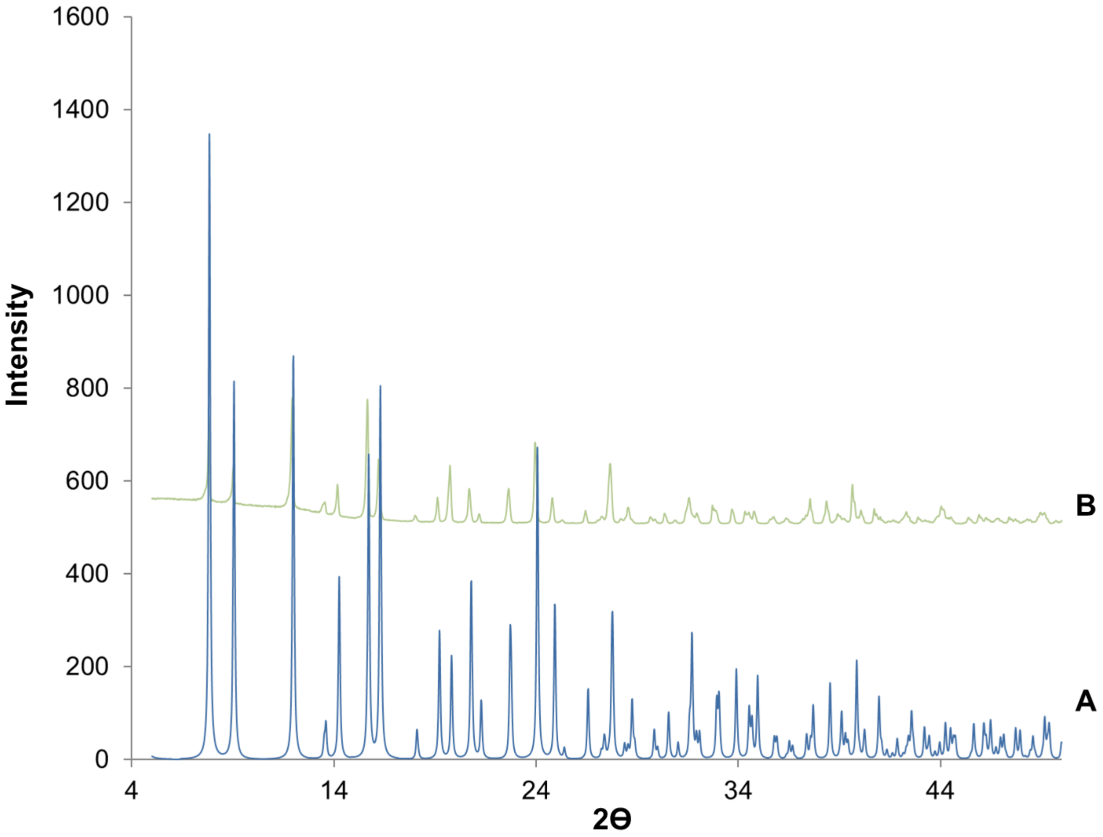

Figure S10. Calculated PXRD of pure UMONT material (a) and PXRD post-exposure to acetic acid in **HOAc20** in a batch set-up (b). Alignment of peaks indicates that the UMON is not degraded by acetic acid.

III. Model Description

1 Characteristics for Langmuirian Adsorption

Langmuirian adsorption characterizes adsorption of species from a bulk phase to a surface. The principle assumptions for Langmurian adsorption are[Allen J. Bard and Larry R. Faulkner, Electrochemical Methods, 2nd Edition, 2001, Wiley and Sons, p 516-519]:

- There are *no interactions between the adsorbate species on the surface.*
- The surface is uniform and featureless.
- The coverage of adsorbates is set by:
  - the concentration of species in the bulk phase
  - the energies of the species in the bulk phase and the adsorbate on the surface (characterized by an equilibrium constant)
  - the number of surface sites available for adsorption.
- At high concentration (activity) of the species in the bulk phase, the *maximum surface coverage* of the adsorbates is monolayer coverage.

2 Rate Expressions for Langmurian Adsorptions

Coverage of the surface is based on the total number of available surface sites Γ^* (mol cm^{-2}). The sites of Γ^* either contain no adsorbate (open) or have an adsorbate, where $\Gamma_{open}(t)$ is the measure of sites open and available for adsorption and $\Gamma_i(t)$ is the coverage of sites by species i adsorbed at time t.

$$\Gamma^* = \Gamma_{open}(t) + \sum_{i=1} \Gamma_i(t) \tag{1}$$

The available (open) sites at time t is:

$$\Gamma_{open}(t) = \Gamma^* - \sum_{i=1} \Gamma_i(t) \tag{2}$$

For the bulk phase a gas, species X_i in present in the bulk gas phase at concentration $[X_i]_g$. The rate of adsorption and desorption of X_i is characterized by rate constants. Species X_i adsorbs to the surface with rate constant $k_{f,i}$ and desorbs with rate constant $k_{b,i}$. The rate of adsorption of species i is

$$\frac{d\Gamma_i(t)}{dt} = k_{f,i}[X_i]_g \Gamma_{open}(t) - k_{b,i}\Gamma_i(t) \tag{3}$$

$$= k_{f,i}[X_i]_g \Gamma^* - k_{f,i}[X_i]_g \sum_{i=1} \Gamma_i(t) - k_{b,i}\Gamma_i(t) \tag{4}$$

For all surface coverages in units of mol cm^{-2} and $[X_i]_g$ the vapor pressure in torr (760 torr = 760 mm Hg = 1.00 atm = 101325 Pa), the units for $k_{f,i}$ and $k_{b,i}$ are torr^{-1}s^{-1} and s^{-1}.

Once the surface coverage is time invariant, $d\Gamma_i(t)/dt = 0$, the system is at equilibrium. The equilibrium constant for species i is $K_i = k_{f,i}/k_{b,i}$

3 Simple Langmuirian Model for Surface Adsorption - One Adsorbate

For a single adsorbate, equation 3 is:

$$\frac{d\Gamma_i(t)}{dt} = k_{f,i}[X_i]_g \Gamma_{open}(t) - k_{b,i}\Gamma_i(t) \tag{5}$$

$$= k_{f,i}[X_i]_g (\Gamma^* - \Gamma_i(t)) - k_{b,i}\Gamma_i(t) \tag{6}$$

$$= k_{f,i}[X_i]_g \Gamma^* - \left(k_{f,i}[X_i]_g + k_{b,i}\right)\Gamma_i(t) \tag{7}$$

3.1 Rate of adsorption for one species

Allow that the initial coverage is $\Gamma_i(t=0)=0$. Then, solved by Laplace transforms, $\Gamma_i(t)$ the time dependent surface concentration of species i is found. The equilibrium constant, $K_i = k_{f,i}/k_{b,i}$.

$$\begin{aligned}\Gamma_i(t) &= \frac{K_i[X_i]_g}{1+K_i[X_i]_g}\left\{1-\exp\left[-\left(k_{f,i}[X_i]_g+k_{b,i}\right)t\right]\right\} && (8)\\ &= \Gamma_{i,\max}\left\{1-\exp\left[-\left(k_{f,i}[X_i]_g+k_{b,i}\right)t\right]\right\} && (9)\end{aligned}$$

$\Gamma_i(t)$ is followed as weight adsorbed with time.

Several critical points are identified. (1) As $t\to\infty$, $\Gamma_i(t\to\infty)\to\frac{K_i[X_i]_g}{1+K_i[X_i]_g}$. (2) As $t\to 0$, $\Gamma_i(t\to 0)\to 0$. The time dependent conditions for the problem are satisfied by (1) and (2). At time $t_{1/2}$ at half maximum coverage, $\Gamma_i\left(t_{1/2}\right)=0.5\Gamma_{i,\max}$, it is noted that $k_f[X]_g=\frac{0.693\Gamma_{\max}}{t_{1/2}}$.

3.1.1 Equilibrium

From the rate expression at a given $[X_i]_g$, equilibrium is achieved as $t\to\infty$ and $d\Gamma_i(t)/dt=0$. From equation 8 as $t\to\infty$, the equilibrium and maximum surface coverage of i, $\Gamma_i(t\to\infty)=\Gamma_{i,\max}$.

$$\lim_{t\to\infty}\Gamma_i(t)=\frac{K_i[X_i]_g}{1+K_i[X_i]_g}=\Gamma_{i,\max} \tag{10}$$

The limiting, final, and maximum weight of adsorbate is $\Gamma_{i,\max}$.

3.1.2 Rate Expression

Rearranging the rate expression equation 8, time dependent rate data are evaluated as

$$\ln\left\{1-\frac{\Gamma_i(t)}{\Gamma_{i,\max}}\right\}=-\left(k_{f,i}[X_i]_g+k_{b,i}\right)t \tag{11}$$

A plot of $\ln\left\{1-\frac{\Gamma_i(t)}{\Gamma_{i,\max}}\right\}$ versus t yields slope $-\left(k_{f,i}[X_i]_g+k_{b,i}\right)$ and intercept of zero.

3.2 The Experiment

The experiment measures total surface coverage $\sum_{i=1}\Gamma_i(t)$ as weight adsorbed. To account for different initial sample weights, the weight fraction adsorbed is reported as $w(t)$, the ratio of weight adsorbed with time normalized by the initial sample weight. The final equilibrium weight fraction is $w_{\max}$.

For a single adsorbate where weight fraction adsorbed is followed with time $w(t)$, time dependent response is then reported.

$$\begin{aligned}w(t) &= \frac{K_i[X_i]_g}{1+K_i[X_i]_g}\left\{1-\exp\left[-\left(k_f[X_i]_g+k_b\right)t\right]\right\} && (12)\\ &= w_{\max}\left\{1-\exp\left[-\left(k_f[X_i]_g+k_b\right)t\right]\right\} && (13)\end{aligned}$$

$$\ln\left\{1-\frac{w(t)}{w_{\max}}\right\}=-\left(k_f[X_i]_g+k_b\right)t \tag{14}$$

Time is in seconds.

The vapor phase concentrations are expressed as vapor pressure. Here torr (mm Hg) are used. Vapor pressure of component i in a mixture of gas phase components is the product of the mole fraction of i in the gas phase x_i and the saturation vapor pressure of component i at the experimental temperature and pressure, $V_{i,sat}$, so that $[X_i]_g=x_iV_{i,sat}$. For water in air at a specified relative humidity $\%RH$, the vapor pressure of water is tabulated. See for example http://www.respirometry.org/calculator/water-vapor-calculators.

For the solvents here, experimental measures of saturation vapor pressures are found as follows.
D_2O/H_2O: https://link.springer.com/article/10.1007/BF00500788, http://www.idc-online.com/technical_references/pdfs
$EtOH/H_2O$ and Acetone/H_2O: doi:10.1088/1742-6596/1369/1/012056
Acetic Acid/H_2O: dx.doi.org/10.1021/ie102105r | Ind. Eng. Chem. Res. 2011, 50, 5795–5805
Saturation vapor pressures pure solvents: https://www.engineeringtoolbox.com/vapor-pressure-d_312.html

3.3 Data Analysis

Data care analyzed as steady state and transient response.

3.3.1 Steady State Data Analysis

Where $w(t)$ is tracked with time, $w_{\max}$ is found as the final weight fraction. If only the final weight fraction is known, then $w_{\max}$ is also known.

From this steady state analysis and equation 12, $K[X]_g$ for the single adsorbate at vapor pressure $[X]_g$ is found.

$$K[X]_g = \frac{w_{\max}}{1 - w_{\max}} \tag{15}$$

Given the vapor pressure of the adsorbate $[X]_g$, K is found, where $K = k_f/k_b$.

The equilibrium constant for adsorption K yields the free energy for adsorption ΔG.

$$\Delta G = -RT \ln K \tag{16}$$

3.3.2 Transient Data Analysis

Where the weight fraction adsorbed $w(t)$ is tracked as a function of time, k_f and k_b are determined. Equilibrium provides the values for $\Gamma_{\max}$, $K[X]_g$, and K, where $K = k_f/k_b$.

From equation 14,

$$\ln\left\{1 - \frac{w(t)}{w_{\max}}\right\} = -\left(k_f [X]_g + k_b\right) t \tag{17}$$

a plot of $\ln\left\{1 - \frac{w(t)}{w_{\max}}\right\}$ versus t yields a slope $-\left(k_f [X]_g + k_b\right)$ or given $w_{\max} = \frac{K_i [X_i]_g}{1+K_i [X_i]_g}$,

$$\ln\left\{1 - \frac{w(t)}{w_{\max}}\right\} = -\left(k_f [X]_g + k_b\right) t \tag{18}$$

$$= -k_f [X]_g \quad 1 + \frac{k_b}{k_f [X]_g}\Bigg) t \tag{19}$$

$$= -k_f [X]_g \quad 1 + \frac{1}{K[X]_g}\Bigg) t \tag{20}$$

$$= -k_f [X]_g \quad \frac{1 + K[X]_g}{K[X]_g}\Bigg) t \tag{21}$$

$$= -\frac{k_f [X]_g}{w_{\max}} t \tag{22}$$

Then,

$$slope = -\frac{k_f [X]_g}{w_{\max}} \tag{23}$$

$[X]_g$ and $w_{\max}$ are known and k_f is determined.

Given K and k_f,

$$k_b = \frac{k_f}{K} \tag{24}$$

A plot of $\ln\left(1 - \frac{w(t)}{w_{\max}}\right)$ vs t has intercept of zero and slope of $-\frac{k_f [X]_g}{w_{\max}}$ where $w_{\max}$ is known. Note that as $w(t)$ approaches $w_{\max}$, the argument of the ln will approach 0 and is undefined.

V. References

[1] A. J. Bard and L. R. Faulkner, Electrochemical Methods: Fundamentals and Applications, 2nd Edition, 2nd ed. Wiley Global Education, 2000, pp. 516–519.

[2] "Water Vapor Calculators," Respirometry.org. http://www.respirometry.org/calculator/water-vapor-calculators (accessed Dec. 06, 2021).

[3] Matsunaga and Nagashima, "Saturation vapor pressure and critical constants of H2O, D2O, T2O, and their isotopic mixtures," International Journal of Thermophysics, vol. 8, no. 6, pp. 681–694, doi: 10.1007/BF00500788.

[4] Maddox, http://www.idc-online.com/technical_references/pdfs/chemical_engineering/Water_properties.pdf

[5] M. S. Makarov and S. N. Makarova, "Heat and mass transfer in the boundary layer during evaporation of aqueous solutions of ethanol and acetone into air and superheated vapor components of solutions," Journalof Physics: Conference Series, vol. 1369, no. 1, p. 012056, Nov. 2019, doi: 10.1088/1742-6596/1369/1/012056.

[6] M. P. Breil, G. M. Kontogeorgis, P. K. Behrens, and M. L. Michelsen, "Modeling of the Thermodynamics of the Acetic Acid-Water Mixture Using the Cubic-Plus-Association Equation of State," Industrial & Engineering Chemistry Research, vol. 50, no. 9, pp. 5795–5805, Mar. 2011, doi: 10.1021/ie102105r.

[7] "Liquids," Vapor Pressures. https://www.engineeringtoolbox.com/vapor-pressure-d_312.html (accessed Dec. 06, 2021).

3.4. Model Equation Development

Consider a surface with a total number of available surface sitesΓ * whereΓ $_{open}$ (t) is the measure of sites open and available for adsorption andΓ (t) is the coverage of sites already coated at time t.

$$\Gamma^* = \Gamma_{open}(t) + \Gamma(t) \tag{25}$$

For adsorption of species X in the gas phase at concentration $[X]_g$ at rate k_f and desorption of adsorbed species at rate k_b, the rate of change for the surface coverage of adsorbate is specified.

$$\begin{aligned}\frac{d\Gamma(t)}{dt} &= k_f[X]_g\Gamma_{open}(t) - k_b\Gamma(t) &(26)\\ &= k_f[X]_g(\Gamma^* - \Gamma(t)) - k_b\Gamma(t) &(27)\\ &= k_f[X]_g\Gamma^* - \Gamma(t)\left[k_f[X]_g + k_b\right] &(28)\end{aligned}$$

Allow that the initial coverage is $\Gamma(t=0)=0$. (We can make a greater than 0 value if needed. $0 \le \Gamma(t) \le \Gamma^*$) Into Laplace space,

$$s\Gamma(s) - \Gamma(t=0) = \frac{k_f[X]_g\Gamma^*}{s} - \left[k_f[X]_g + k_b\right]\Gamma(s) \tag{29}$$

Substituting $\Gamma(t=0)=0$ and rearranging,

$$\begin{aligned}\Gamma(s)\left(s + k_f[X]_g + k_b\right) &= \frac{k_f[X]_g\Gamma^*}{s} &(30)\\ \Gamma(s) &= \frac{k_f[X]_g\Gamma^*}{s\left(s + k_f[X]_g + k_b\right)} &(31)\end{aligned}$$

By partial fractions

$$\begin{aligned}\Gamma(s) &= \frac{k_f[X]_g\Gamma^*}{s\left(s + k_f[X]_g + k_b\right)} = \frac{A_1}{s} + \frac{B_1}{s + k_f[X]_g + k_b} &(32)\\ k_f[X]_g\Gamma^* &= A_1\left(s + k_f[X]_g + k_b\right) + B_1 s &(33)\end{aligned}$$

At nodes

$$\begin{aligned}\lim_{s\longrightarrow 0} k_f[X]_g\Gamma^* &= A_1\left(k_f[X]_g + k_b\right) &(34)\\ A_1 &= \frac{k_f[X]_g\Gamma^*}{k_f[X]_g + k_b} &(35)\\ \lim_{s\longrightarrow -\left(k_f[X]_g + k_b\right)} k_f[X]_g\Gamma^* &= -B_1\left(k_f[X]_g + k_b\right) &(36)\\ B_1 &= -\frac{k_f[X]_g\Gamma^*}{k_f[X]_g + k_b} = -A_1 &(37)\end{aligned}$$

Allow $K = k_f/k_b$.Then

$$\begin{aligned}\Gamma(s) &= \frac{A_1}{s} + \frac{B_1}{s + k_f[X]_g + k_b} &(38)\\ \Gamma(s) &= \frac{K[X]_g}{1 + K[X]_g}\left\{\frac{1}{s} - \frac{1}{s + k_f[X]_g + k_b}\right\} &(39)\\ \Gamma(t) &= \frac{K[X]_g}{1 + K[X]_g}\left\{1 - \exp\left[-\left(k_f[X]_g + k_b\right)t\right]\right\} &(40)\end{aligned}$$

from equation 40, test of specification:

$$\lim_{t \longrightarrow 0} \Gamma\left(t\right) \quad \longrightarrow \quad 0 \ \checkmark \tag{41}$$

$$\lim_{t \longrightarrow \infty} \Gamma\left(t\right) \quad \longrightarrow \quad \frac{K\left[X\right]_g}{1 + K\left[X\right]_g} \ \checkmark \tag{42}$$

where the second expression is the Langmuirian isotherm.

IV. Data fitting for H_2O and binary systems

Data were collected as weight adsorbed with time. Weights are normalized by the initial sample weight, *w(t)*. A final weight adsorbed is expressed as a fraction w_{max} or percent w_{max}%. For most samples, more than one replicate was collected, specified as number in sample n. Where n > 1, the data were averaged and the average data were fit. Shown are the fits for each of the water (RH) and mixtures of water and another solvent. The quality of the fit varies with the system. In each of the following plots, the rightmost axis is represented by the orange data points and the grey points represent the model. The blue points lie on the leftmost axis.

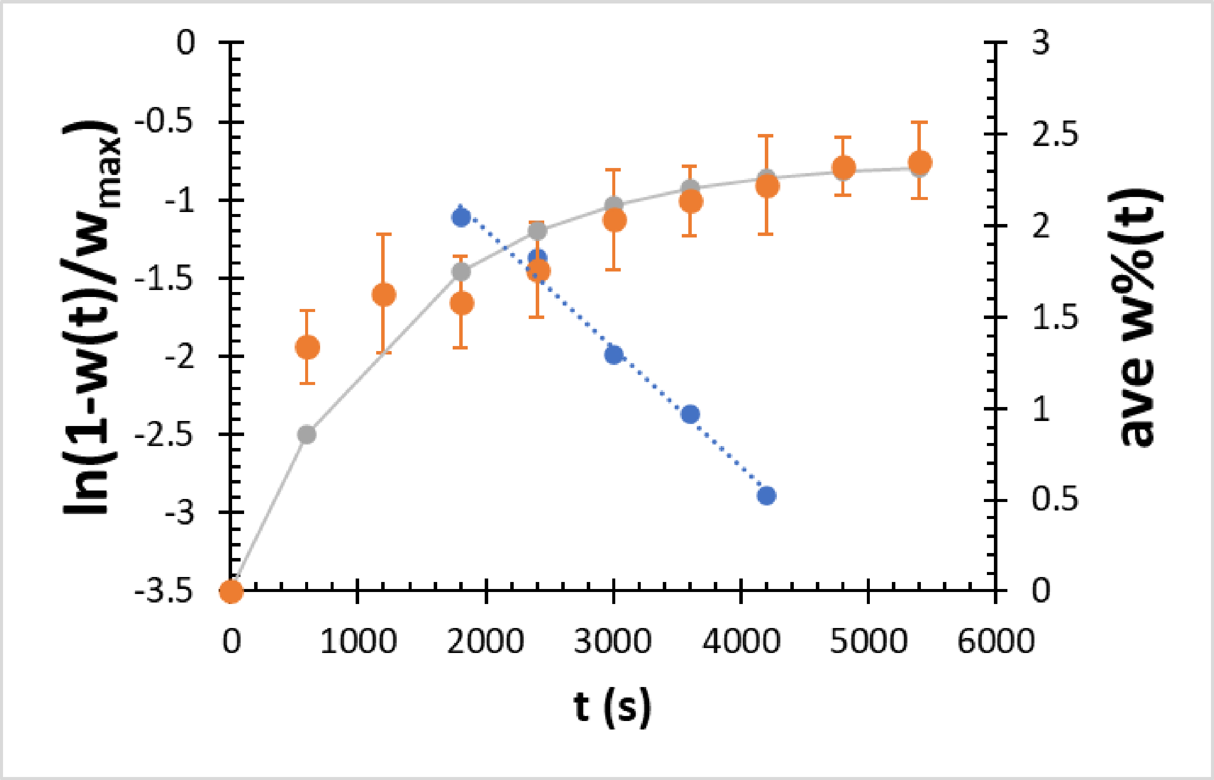

Figure S11. Modeled fitting of **D_2O 100**, where n=3 and the fit has an R^2 value of 0.989.

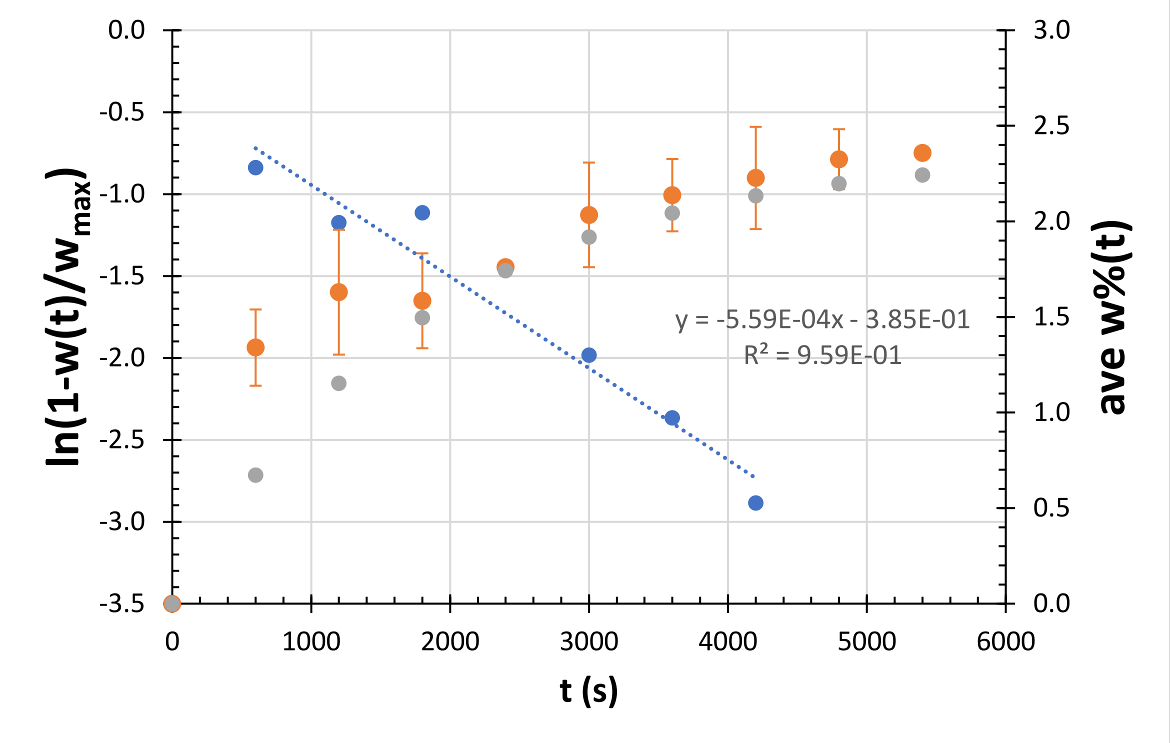

Figure S12. Modeled fitting of **D_2O 90**, where n=3 and the fit has an R^2 value of 0.959.

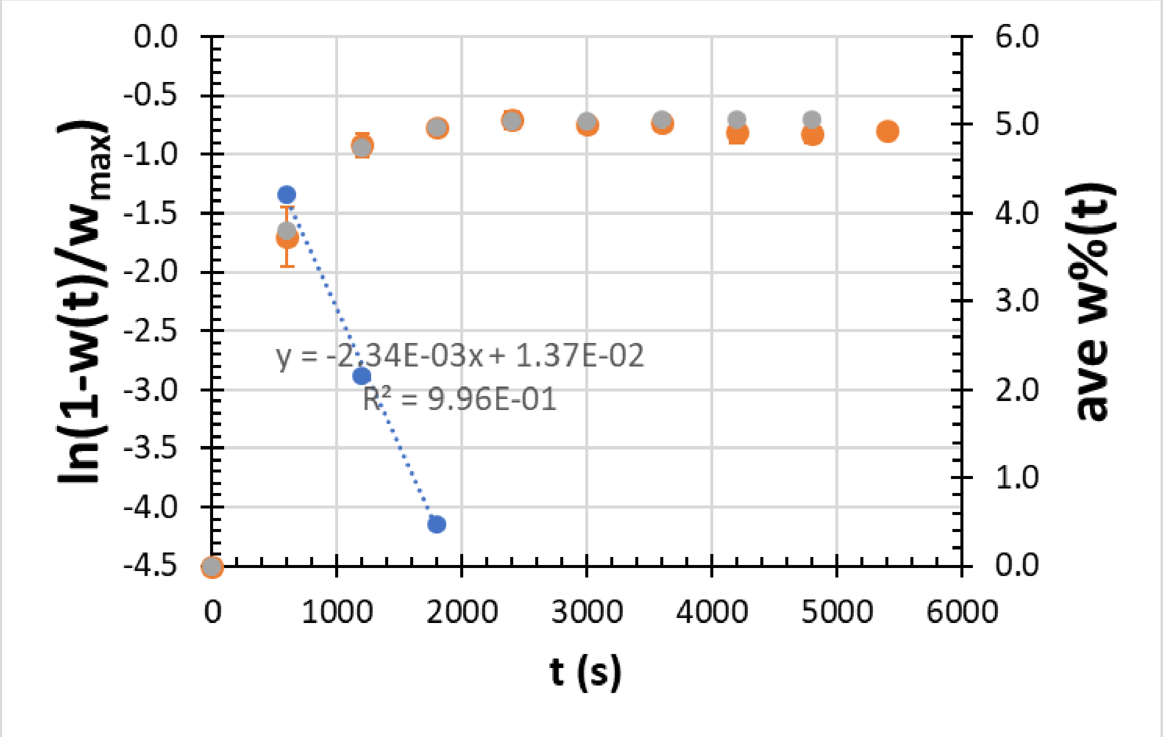

Figure S13. Modeled fitting of **D_2O 50**, where n=3 and the fit has an R^2 value of 0.996.

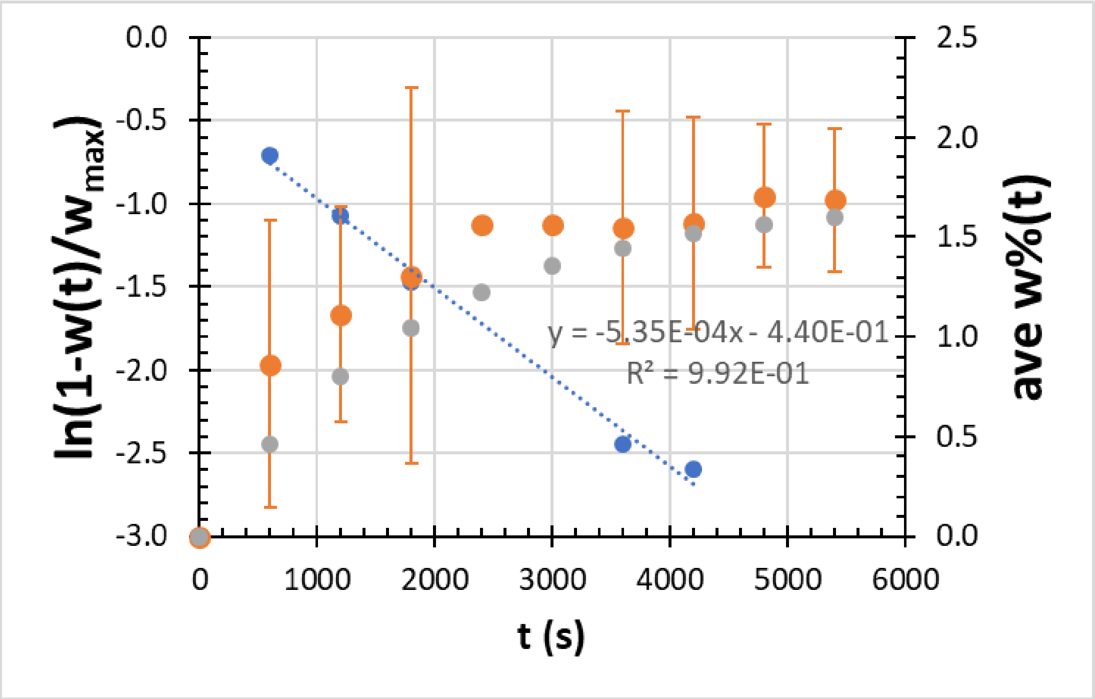

Figure S14. Modeled fitting of **ETOH 90**, where n=3 and the fit has an R^2 value of 0.992

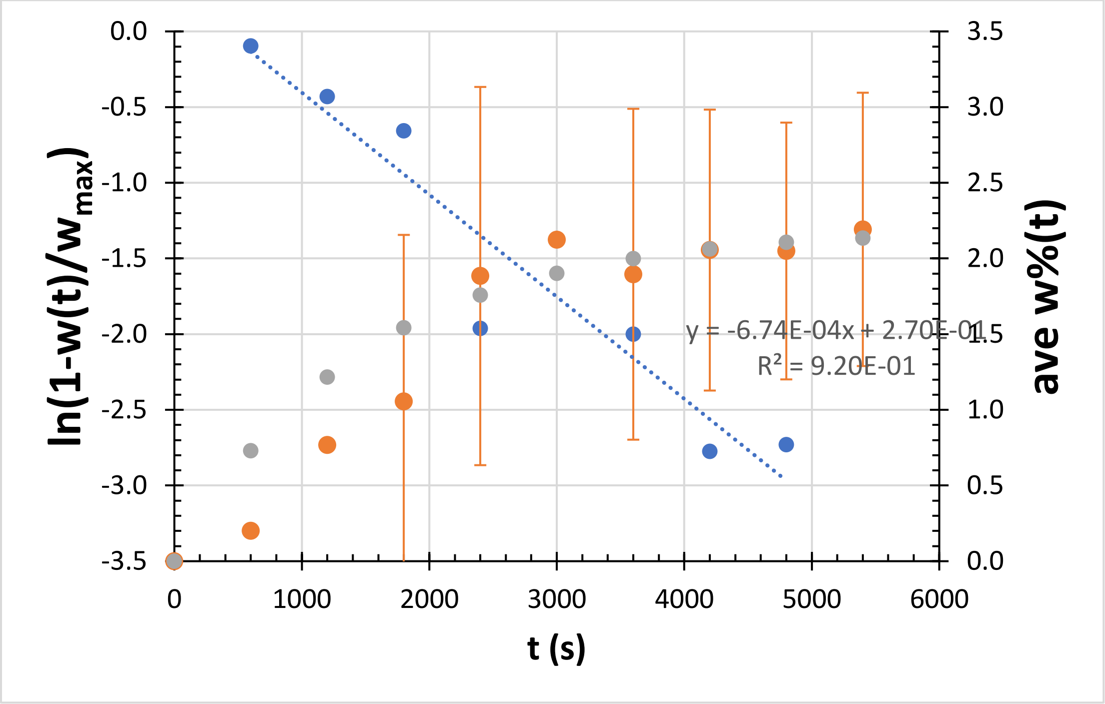

Figure S15. Modeled fitting of **ETOH 80**, where n=3 and the fit has an R^2 value of 0.920

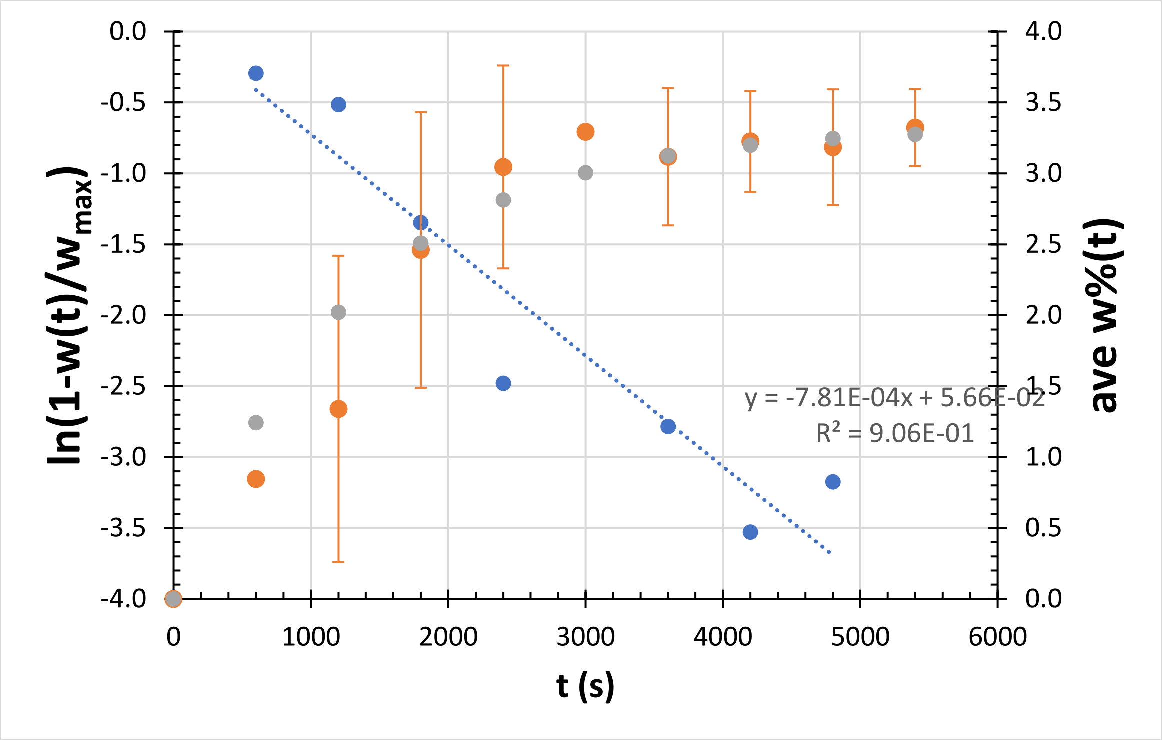

Figure S16. Modeled fitting of **ETOH 50**, where n=3 and the fit has an R^2 value of 0.906.

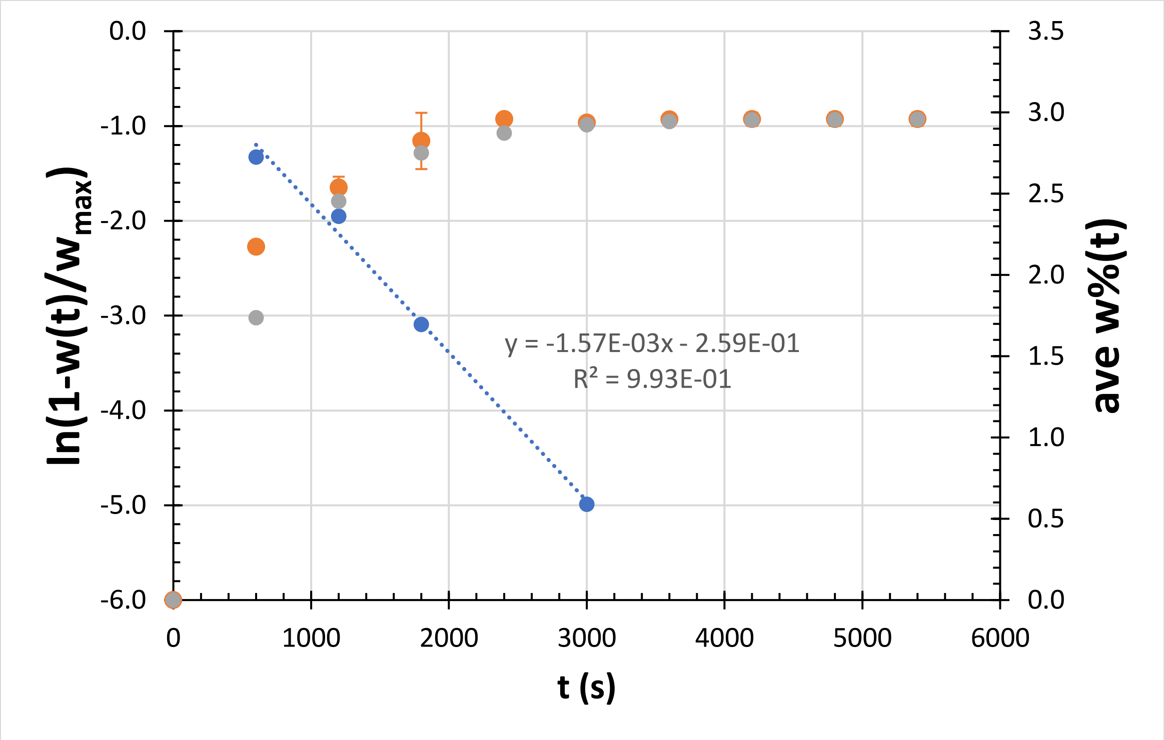

Figure S17. Modeled fitting of **ETOH 10**, where n=3 and the fit has an R^2 value of 0.993.

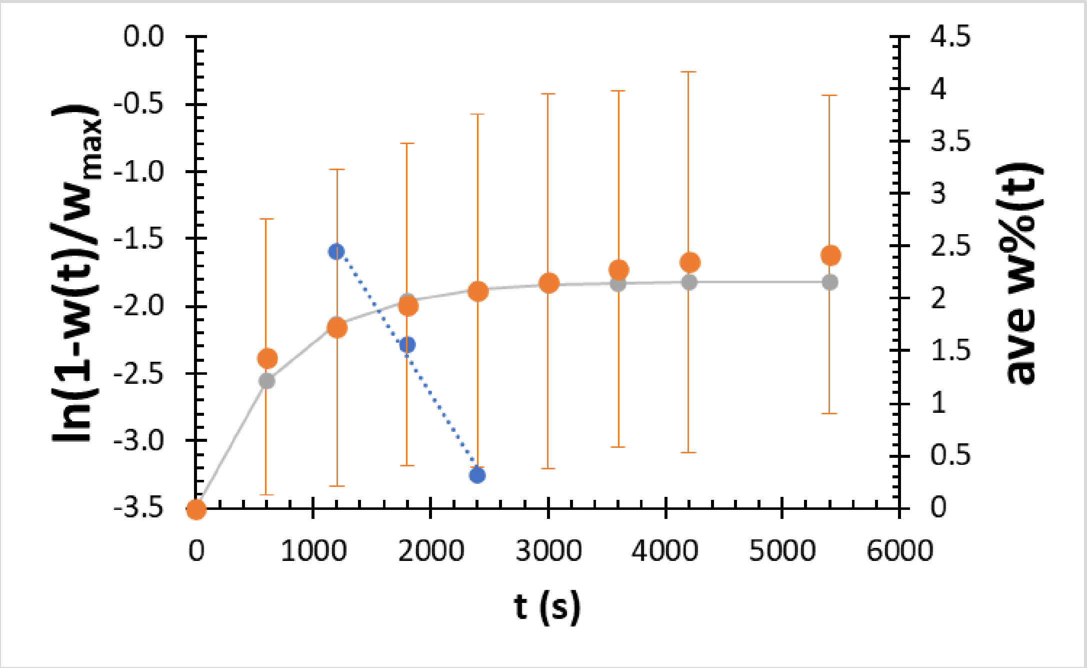

Figure S18. Modeled fitting of **ACE 50**, where n=3 and the fit has an R^2 value of 0.990.

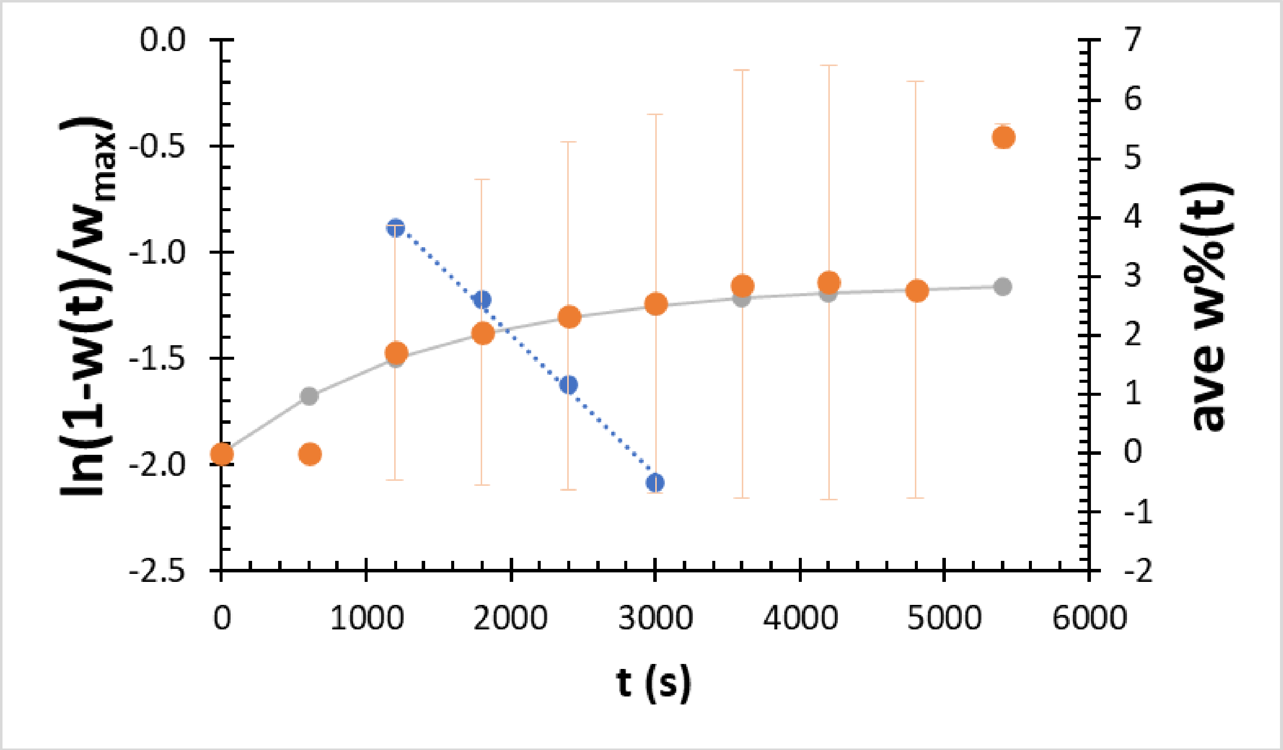

Figure S19. Modeled fitting of **HOAc 20** experimental data, where n=2 and the fit has an R^2 value of 0.995.
